# The global gene expression outline of the bovine blastocyst: reflector of environmental conditions and predictor of developmental capacity

**DOI:** 10.1186/s12864-021-07693-0

**Published:** 2021-06-03

**Authors:** Dessie Salilew-Wondim, Dawit Tesfaye, Franca Rings, Eva Held-Hoelker, Dennis Miskel, Marc-Andre Sirard, Ernst Tholen, Karl Schellander, Michael Hoelker

**Affiliations:** 1 Institute of Animal Sciences, Animal Breeding, University of Bonn, Endenicher Allee 15, 53115 Bonn, Germany; 2 Animal Reproduction and Biotechnology Laboratory, Department of Biomedical Sciences, Colorado State University, 3105 Rampart Rd, CO 80521 Fort Collins, USA; 3 Centre de Recherche en Reproduction, Développement et Santé Intergénérationnelle, Faculté des sciences de l’agriculture et de l’alimentation, INAF, Pavillon des services, Université Laval (Québec), G1V 0A6, Quebec City, Canada; 4 Department of Animal Science, Biotechnology & Reproduction in farm animals, University of Goettingen, Burckhardtweg 2, 37077 Goettingen, Germany

**Keywords:** Bovine, Embryo, Transcriptome, Pregnancy

## Abstract

**Background:**

Morphological evaluation of embryos has been used to screen embryos for transfer. However, the repeatability and accuracy of this method remains low. Thus, evaluation of an embryo’s gene expression signature with respect to its developmental capacity could provide new opportunities for embryo selection. Since the gene expression outline of an embryo is considered as an aggregate of its intrinsic characteristics and culture conditions, we have compared transcriptome profiles of in vivo and in vitro derived blastocysts in relation to pregnancy outcome to unravel the discrete effects of developmental competence and environmental conditions on bovine embryo gene expression outlines. To understand whether the gene expression patterns could be associated with blastocyst developmental competency, the global transcriptome profile of in vivo (CVO) and in vitro (CVT) derived competent blastocysts that resulted in pregnancy was investigated relative to that of in vivo (NVO) and in vitro (NVT) derived blastocysts which did not establish initial pregnancy, respectively while to unravel the effects of culture condition on the transcriptome profile of embryos, the transcriptional activity of the CVO group was compared to the CVT group and the NVO group was compared to the NVT ones.

**Results:**

A total of 700 differentially expressed genes (DEGs) were identified between CVO and NVO blastocysts. These gene transcripts represent constitutive regions, indel variants, 3′-UTR sequence variants and novel transcript regions. The majority (82%) of these DEGs, including gene clusters like ATP synthases, eukaryotic translation initiation factors*,* ribosomal proteins, mitochondrial ribosomal proteins, NADH dehydrogenase and cytochrome c oxidase subunits were enriched in the CVO group. These DEGs were involved in pathways associated with glycolysis/glycogenesis, citrate acid cycle, pyruvate metabolism and oxidative phosphorylation. Similarly, a total of 218 genes were differentially expressed between CVT and NVT groups. Of these, 89%, including *TPT1*, *PDIA6*, *HSP90AA1* and *CALM,* were downregulated in the CVT group and those DEGs were overrepresented in pathways related to protein processing, endoplasmic reticulum, spliceasome, ubiquitone mediated proteolysis and steroid biosynthesis. On the other hand, although both the CVT and CVO blastocyst groups resulted in pregnancy, a total of 937 genes were differential expressed between the two groups. Compared to CVO embryos, the CVT ones exhibited downregulation of gene clusters including ribosomal proteins, mitochondrial ribosomal protein, eukaryotic translation initiation factors, ATP synthases, NADH dehydrogenase and cytochrome c oxidases. Nonetheless, downregulation of these genes could be associated with pre and postnatal abnormalities observed after transfer of in vitro embryos.

**Conclusion:**

The present study provides a detailed inventory of differentially expressed gene signatures and pathways specifically reflective of the developmental environment and future developmental capacities of bovine embryos suggesting that transcriptome activity observed in blastocysts could be indicative of further pregnancy success but also adaptation to culture environment.

**Supplementary Information:**

The online version contains supplementary material available at 10.1186/s12864-021-07693-0.

## Introduction

Selecting transferable embryos that could sustain pregnancy has been a challenge in the field of assisted reproductive technology. Indeed, in humans, non-invasive selection strategies based on morphological evaluation have been used to select the best embryos. These grading techniques take into account the appearance of the cytoplasm, size and shape of blastomeres, embryo fragmentation [1], number of cleavages (even or uneven) [1, 2], cleavage kinetics [3], blastomere multinucleation [4–7] or a combination of these [8]. Morphological classification of bovine embryos prior to transfer to recipient animals represents the common practice. Usually, the bovine embryo is morphologically classified as grade 1 (excellent or good), grade 2 (regular), grade 3 (poor) or grade 4 (dead or degenerating embryos). Grade 1 in vivo derived embryos are eligible for international trade as they are suggested to be viable and to survive freeze/thawing well, whereas grade 2 and 3 are recommended for transfer fresh into recipient animals [9]. Although, morphological classification methods have substantial value, repeatability and accuracy of morphological parameters are generally fraught with errors due to the subjectivity of classification. Moreover, even embryos graded as low quality might be able to develop to term [10] suggesting that selecting embryos based on morphological appearance has potential drawbacks. Furthermore, preimplantation genetic screening by testing for chromosomal abnormalities as well as activity of genes related to metabolism has been used for selecting developmentally competent embryos [11]. Therefore, an embryo screening method that provides complete information about an embryo’s intrinsic characteristics, such as its metabolism and its gene expression pattern could be an alternative to subjective analysis during selection. In that regard, it would be interesting to identify and characterize molecular signatures that are associated with an embryo’s developmental capacity. For instance, understanding the expression of genes that could cause termination of pregnancy by affecting embryonic genome activation, blastocyst formation, embryo elongation or secretion of interferon-tau [12] could be one step forward to identify molecular markers useful for classifying an embryo’s individual developmental potential. This, however, could be even more relevant for in vitro derived embryos since their developmental capacity might be more compromised by its non-physiological preimplantation environment factors like culture media, in vitro culture conditions such as oxygen level, pH, temperature, humidity and others. Indeed, oxygen tension [13], heat stress [14] as well as the principal formulation of the culture medium itself [15, 16] were found to affect the developmental competence of bovine embryos by altering the expression of genes associated with pluripotency, trophectoderm formation and apoptosis. Subsequently, suboptimal in vitro culture conditions are suggested to hinder embryonic developmental competence by altering the expression profile or epigenetic landscape of genes associated with embryonic development. With this respect, a previous study has shown alterations in expression of 134 transcripts at 4-cell stage and 97 transcripts in 8-cell stage embryos derived from in vitro compared to the in vivo derived ones [17], indicating the impact of the in vitro culture environment on the gene expression patterns of the bovine embryo. Similarly, a stage specific exposure of bovine embryos to in vitro culture condition before or after embryonic genome activation has unravelled alterations in expression of genes involved in lipid metabolism and oxidative phosphorylation [18]. Moreover, several candidate genes and large scale transcriptome profile analysis approaches [18–24] and DNA methylation studies [25–30] have proven the effect of culture conditions on gene expression patterns and epigenetic profiles in the resultant blastocysts. Collectively, it is generally accepted that the transcriptome profile of the bovine blastocyst depends on the culture conditions of the in vitro culture. However, all studies comparing the gene expression signature of bovine embryos derived from different culture environments so far have not considered the developmental capacity of the individual embryos analysed. With this respect, it is well known that in vivo derived embryos develop to a much higher extent into healthy offspring compared to in vitro derived ones, it is questionable to compare populations of in vivo and in vitro derived embryos containing contrasting proportions of embryos bearing high and low developmental capacity. Therefore, doing so indicates not only the effect of the environment but also the impact of contrasting proportions of competent embryos analysed in these studies. Thus, the consequence of contrasting intrinsic embryo qualities has been measured and interpreted wrongly as reflecting the environment.

The outline of an embryo’s transcriptome profile could be used to predict the embryo’s individual developmental capacity. This is based on the hypothesis that, unlike the non-competent ones, competent in vivo or in vitro derived developmentally competent embryos are endowed with typical molecular signatures necessary to support further development. Thus, investigating the association between embryonic developmental competence and their molecular signatures could provide an opportunity to generate molecular markers that could be used as predictors of embryonic developmental competence. Earlier, we and others have demonstrated the correlation between gene expression patterns of in vitro or in vivo produced bovine blastocyst with their developmental competence [31–35]. However, for some studies [31–33], the numbers of probes (including the controls) incorporated in the microarray platform were few. Other studies, [34, 35] were focused on the gene expression patterns in relation to female embryo developmental competency. Moreover, previous conclusions drawn about the gene expression of developmentally competent in vivo and in vitro derived bovine embryos was done indirectly by performing meta-analysis, but no direct comparison between competent embryos derived from different developmental environments has been conducted so far. Thus, molecular signatures which are predictors of developmental capacity without interfering effects of the given developmental environment have not been specifically determined so far. Therefore, further studies correlating the gene expression signature with future developmental capacity are necessary to further enhance our predictive power in determining the developmental capacity of bovine embryos.

Collectively, it is unquestionable that the gene expression outline of the bovine embryo partially reflects its culture environment during early development as well as predicting its future developmental capacity. The latter in turn, might be partially affected by the culture environment as well as predetermined by the embryo’s intrinsic quality independent from the culture environment. Therefore, the principal aim of the present study was first to unravel specific molecular signatures predictive for developmental capacity and to identify those molecules specifically reflective of culture/environmental conditions. These insights might be beneficial for selecting the best embryo for transfer but also to unravel environmental conditions interactively affecting the expression outline of genes related to viability.

## Materials and methods

### Experimental design

**Fig. 1:**
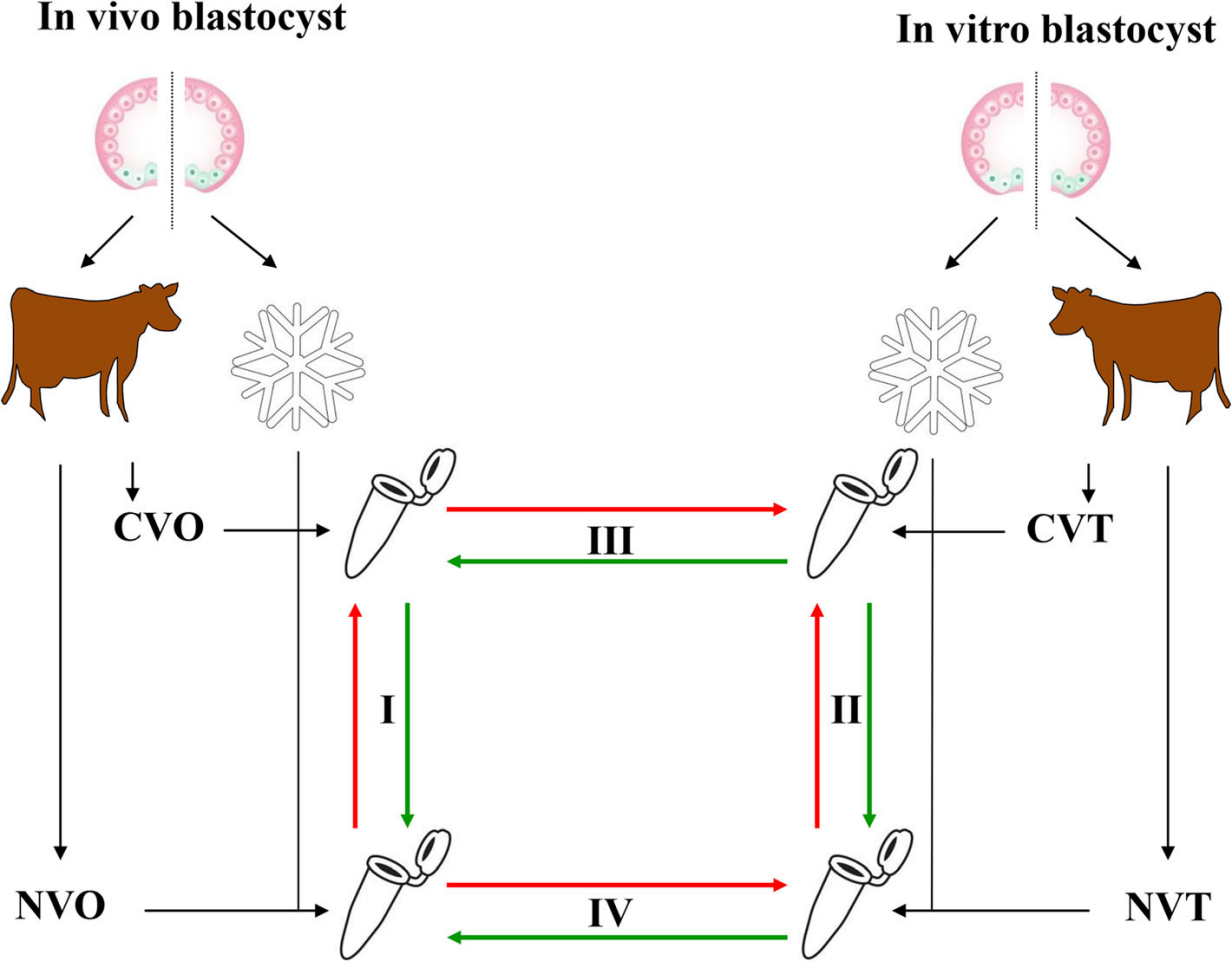
The experimental design used for comparative gene expression analysis in embryo biopsies. Numbers I, II, III and VI indicate comparisons with regard to the global gene expression profile between competent (CVO) and non-competent (NVO) in vivo derived blastocysts, competent (CVT) and non-competent (NVT) in vitro derived blastocysts, competent in vitro derived blastocysts (CVT) and competent in vivo derived blastocysts (CVO) as well as non-competent in vitro derived blastocysts (NVT) and non-competent in vivo derived blastocysts (NVO), respectively

To unravel the proposed questions of the present study, four gene expression studies were conducted (Fig. 1). To unravel the gene expression patterns specifically caused by contrasting developmental capacities which are typical for the in vivo derived embryos, I) the transcriptome profile of competent in vivo derived embryos (CVO) was compared with the profile of non-competent in vivo derived embryos (NVO). Likewise, to unravel the gene expression signature specifically caused by contrasting developmental capacities typical for vitro derived embryos, II) the transcriptome profile of competent in vitro derived embryos (CVO) was compared with the profile of non-competent in vitro derived embryos (NVO). To explore the gene expression pattern of bovine embryos being specifically a consequence of contrasting culture conditions without conflictive with developmental capacity, III) the transcriptome profile of competent in vitro derived embryos (CVT) was compared with the profile of competent in vivo derived embryos (CVO). Finally, to explore the gene expression pattern of bovine embryos caused by contrasting culture conditions that conflict with developmental capacity, IV) the gene expression profile of non-competent in vitro derived embryos (NVT) was compared with non-competent in vivo derived embryos (NVO) ones.

### Animal handling

Animal handling for collection of in vivo derived embryos and transfer of both in vivo and in vitro derived embryos to synchronized recipients was carried out in accordance with the German Law of Protection (TierSchG & TierSchVersV). All experimental protocols performed on cows in this study were approved by the state office for Nature, Environment, and Consumer Protection of North Rhine-Westphalia, Germany (Landesamt für Natur, Umwelt und Verbraucherschutz Nordrhein-Westfalen, Deutschland) under license number 84–02.04.2014.A499. All experiments were performed in accordance with relevant guidelines and regulations and adheres to the ARRIVE guidelines.

### In vitro embryo production (IVP)

In vitro embryo production was performed using oocytes collected from abattoir-derived ovaries. The bovine ovaries were transported to the laboratory (Campus Frankenforst) of the University of Bonn in a thermo flask containing 0.9% (w/v) saline solution. Upon arrival, the ovaries were washed three times with 0.9% saline solution. Afterwards, cumulus-oocyte complexes (COCs) were aspirated from 2- to 8 mm-diameter follicles. The COCs were then in vitro matured, in vitro fertilized and in vitro cultured as indicated previously [31]. The developmental rates were recorded, and day 7 blastocysts were collected for transfer.

### In vivo embryo production

Simmental heifers were used in in vivo embryo production and embryo transfer. All experimental animals were handled and managed according to the rules and regulations of the German law of animal protection at Campus Frankenforst of the University of Bonn. The procedures of in vivo embryo production was performed as indicated previously [32, 33]. Briefly, Simmental heifers were pre-synchronized using intramuscular administration of 500 mg of the prostaglandin F_2α_ (PGF2α) analogue cloprostenol (Estrumate; Munich, Germany) twice within 11 days and followed by 0.02 mg GnRH-analogue buserelin (Intervet, Boxmeer, The Netherlands) administration after 2 days of each of PGF2a administration. Twelve days after the second GnRH administration, 8 consecutive FSH-injections over 4 days in decreasing doses was performed followed by two PGF_2α_ treatments, 60 and 72 h after the initial FSH. Ovulation was induced by administration of 0.02 mg buserelin and was followed by three inseminations at 12 h intervals. Embryos were flushed 7 days after insemination.

### Blastocyst biopsy transfer

Blastocyst biopsying and transfer was performed as reported previously [31, 32]. Briefly, a 40–50% portion containing both inner cell mass (ICM) and trophectoderm (TE) was biopsied from each blastocyst and snap frozen in cryo-tubes, containing lysis buffer [0.8% Igepal (Sigma-Aldrich, MO, USA), 40 U ml^**−** 1^ RNasin (Promega, WI, USA), 5 mM dithiothreitol) for further analysis. The remaining 50–60% portion of each blastocyst was in vitro cultured in Charles Rosenkrans 1 medium supplemented with amino acid for 2 h and transferred to synchronized **Simmental** heifers. A total of 69 in vivo derived and 59 in vitro derived blastocyst biopsies were transferred to 128 recipient heifers.

### Pregnancy diagnosis and embryo biopsy categorization

Pregnancy diagnosis was performed on days 28 and 42 using ultrasonography (Pie Medical, 5 MHz) and at day 90 by rectal palpation. Following this, in vivo and in vitro derived embryo biopsies taken from those blastocysts which sustained pregnancy until 90 days of gestation were classified as competent in vivo derived (CVO) and competent in vitro derived (CVT) blastocysts, respectively. Similarly, embryo biopsies taken from those blastocysts which did not initiate initial pregnancy were classified as non-competent in vivo derived (NVO) and non-competent in vitro derived (NVT) blastocysts, respectively.

### RNA isolation from embryo biopsies

Total RNAs was isolated from each blastocyst biopsy group (CVO, CVT, NVO, and NVT) in four independent replicates. Each replicate consists of 5 biopsies and RNA isolation was performed using the PicoPure RNA isolation kit (Arcturs, Munich, Germany) following the manufacturer’s protocol. Briefly, each embryo biopsy was incubated with 20 μl extraction buffer at 42° for 30 min. Biopsies from the same group were pooled. After adding 1 volume 70% ethanol, the samples were loaded into the pre- conditioned purification column. The RNA was bound to the column by centrifugation of the samples at 1057 rpm for 2 min, followed by a centrifugation step at 13500 rpm for 30 s. The samples were washed using wash buffers and on column DNase treatment was performed using RNase-fee DNase I (Qiagen, CA, USA). After subsequent steps, the RNA was eluted in 12 μl elution buffers. The quality and concentration of RNA and was evaluated using NanoDrop 8000 Spectrophotometer.

### RNA amplification and array hybridization

The total RNA samples were subjected two rounds of amplification to generate amplified anti-sense RNA using the RNA amplification HS kit (Applied Biosystems). The amplified RNA was eluted in 30 μl of elution buffer and the quality and quantity of amplified RNA samples was evaluated using the NanoDrop 8000 Spectrophotometer. Two microgram of amplified RNA from each sample (CVO, CVT, NVO and NVT) was mixed with either 1 μl of Cy-3 or Cy-5 ULS fluorescent labelling kit (Kreatech Diagnostics, Amsterdam, Netherlands) and incubated at 85 °C for 30 min. Unincorporated Cy-3 and Cy − 5 dyes were removed using the PicoPure RNA extraction kit (Applied Biosystems). Following this, sample mixing was performed following the outline of the experimental design (Fig. 1). Samples were mixed with 157.6 μl hybridization cocktail and incubated at 95 °C for 3 min and at 37 °C for 30 min. Afterwards, 65 μl of Agilent-CGHBlock was added to each sample and transferred onto the EmbryoGENE bovine microarray slides. Each slide contains four arrays, and each array consists of 45,000 probes. The slides were then incubated for 40 h at 65 °C in the hybridization oven. At the end of hybridization, the slides were sequentially washed for 10 min in 2x SSC plus 0.1% SDS, 5 min each in 0.2xSSC and 0.1% SSC buffers, 1 min each in water and isopropanol. Array hybridization was done in a dye-swap design (technical replicates) and for each sample three independent replicates were performed. A total of 24 hybridizations were done for 4 experiments.

### Array image capture and array data analysis

The arrays were scanned using Axon GenePix 4000B scanner and the images of the array were analysed using GenePix Pro analysis software (version 5.0) (Axon Instruments, Foster City, CA) as indicated previously [32]. Briefly, subtract and offset method was used to correct the array background [36] and LOESS and scale-normalization methods were used to normalize differences within array variations [37, 38] and between the arrays, respectively. A mean log_2_ transformed value of (Cy5/Cy3) was calculated from three replicates and the respective dye-swaps to obtain one value per target. Differentially expressed genes were identified using linear models for microarray data [39]. Genes with average log_2_ expression value > 0.65 and ≤ − 0.65 fold change and *p* < 0.05 and adjusted *p* value (FDR) < 0.2 were considered as differentially expressed genes**.**

## Results

A total of 59 biopsied in vitro derived and 69 biopsied in vivo derived embryos were transferred to synchronized Simmental heifers. Of these, 17 (25.4%) biopsied in vitro derived embryos ended up in a stable pregnancy at day 90. Similarly, 15 (24.6%) biopsied in vivo derived embryos were ended up in a stable pregnancy at day 90. Embryos that did not end up in pregnancy at day 28 days of gestation were classified as non-competent embryos and those which resulted in pregnancy at least until day 90 of the gestation period were classified as developmentally competent. To get insight into specific differences with regard to the gene expression outline caused by contrasting viabilities (competent vs. non-competent blastocysts derived from the same environment) as well as caused specifically by contrasting environments (in vitro vs. in vivo derived embryos of equal developmental capacity), the blastocyst biopsies were classified based on pregnancy outcome of the corresponding counterparts as competent in vivo blastocyst (CVO), non-competent in vivo blastocyst (NVO), competent in vitro blastocyst (CVT) and non-competent in vitro blastocyst (NVT).

### Molecular signatures predicting the developmental capacity of in vivo derived bovine embryos

**Fig. 2:**
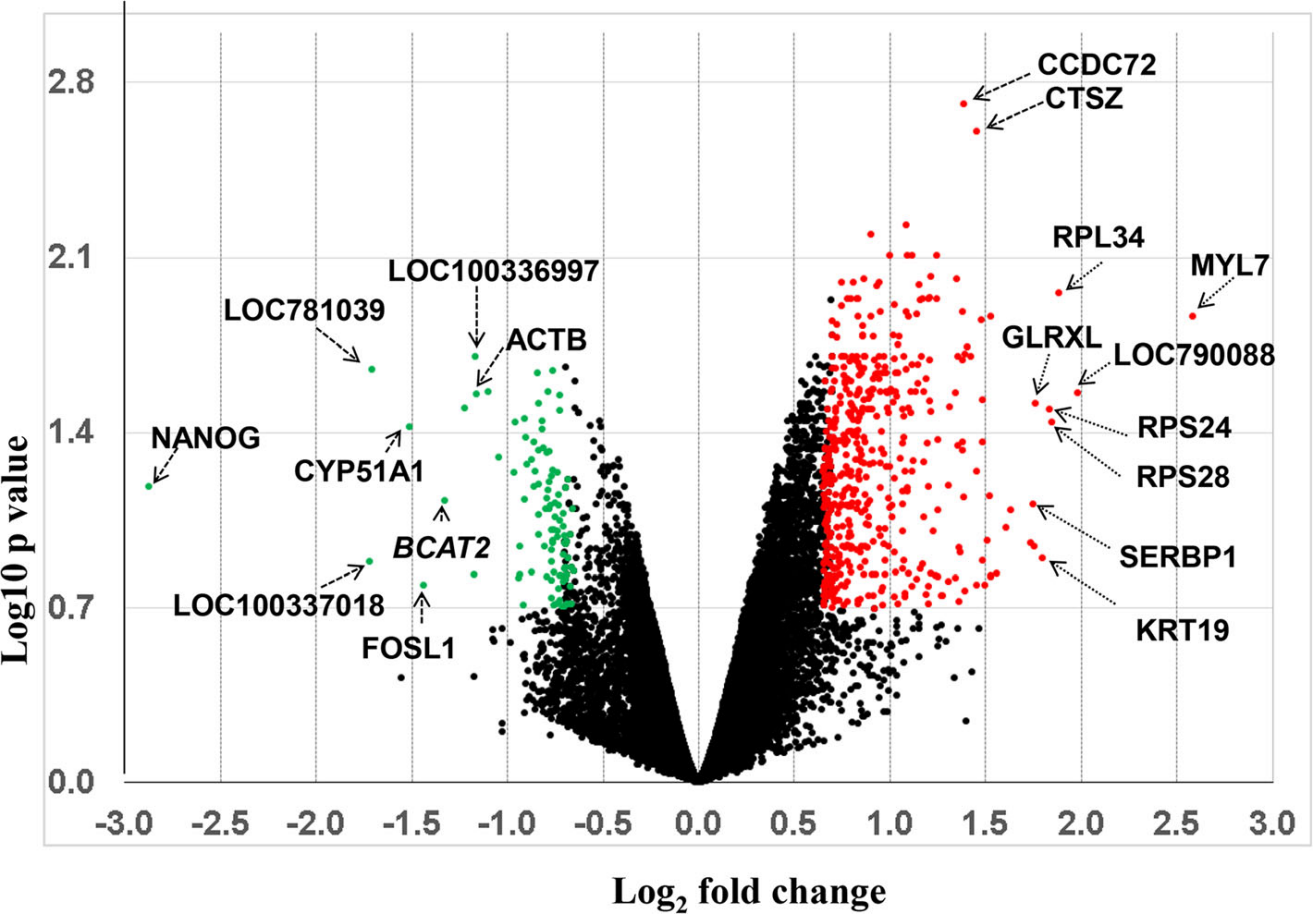
Molecular signature associated with the developmental capacity of in vivo derived embryos. Volcano plot demonstrating differentially expressed genes between CVO and NVO blastocysts. Red and green dots indicate up and downregulated genes, respectively in CVO compared to NVO blastocysts. Transcripts highly significant up or downregulated are indicated with arrows

A total of 766 probes associated with 700 gene transcripts were identified to be differentially expressed between competent in vivo derived embryos (CVO) and non-competent in vivo derived embryos (NVO), (Fig. 2, Table 1). The expression pattern of 634 differentially expressed genes (DEGs) including *RPL34, RPS28, RPS24, KRT19, GLRXL* and *SERBP1* transcripts were significantly increased whereas expression of 132 gene transcripts including *NANOG, CYP51A1, TNIP2, BCAT2, FOSL1* and *ACTB* was significantly decreased in embryos of the CVO group (Fig. 2, Supplemental Table S1). Since the EmbryoGENE microarray (Agilent-028298: Bovine Embryo and Splice Transcriptome microarray) is enriched with annotated genes, uncharacterized transcribed regions, embryo specific indel type variants, alternative 3′UTR events (genes) and pseudo genes [40], we took advantage of this opportunity to investigate the proportion of these gene expression features. Accordingly, a total of 21 (0.06%) and 188 (24.4%) DEGs represented pseudo genes and novel transcripts (NTRs), respectively (Table 1) and a total of 29 genes (3.8% of all DEGs) including *ALDH3A2, TFAP2C, UBE3B, NET1, SNX16, SLC35E3, MAML2, CDYL* and *CYP51A1* represented alternative 3-′UTR events (Tale 1, Table 2). Similarly, a total of 44 differential expressed transcripts including *PLAC8, PRDX5*, *MYL7* and *MYL6* represented gene variants (Table 3). Strikingly, the expression trends of six out of ten MYL6 variants were upregulated in embryos of CVO compared to the NVO group.

**Table 1:** Preferentially expressed probes in CVO and NVO groups

	Enriched in CVO	Enriched in NVO	Total
Constitutive	422	74	506
Novel gene transcribed regions; evidence: embryonic ESTs	158	30	188
Alternative 3′UTR events (genes)	10	19	29
Indel type splice variants	35	9	44
Pseudo genes	23	3	26
Total differentially expressed probes	635	131	766

**Table 2:** Differentially expressed gene variants between CVO and NVO groups

Probe ID	Target ID	Gene symbol	Expression patterns
EMBV3_33164	XM_002697258	*LOC100337465*	**↑**
EMBV3_19607	NM_001101984	*ALDH3A2*	**↑**
EMBV3_20670	NM_001075509	*TFAP2C*	**↑**
EMBV3_15124	XM_002694536	*UBE3B*	**↑**
EMBV3_11386	NM_001034296	*NET1*	**↑**
EMBV3_08558	XM_002692833	*SNX16*	**↑**
EMBV3_30354	XM_002692428	*SRXN1*	**↑**
EMBV3_29131	NM_001098069	*CASC3*	**↑**
EMBV3_28415	NM_174361	*IMPA1*	**↑**
EMBV3_04016	NM_001101989	*TMEM144*	**↑**
EMBV3_23797	NM_001102052	*SKP2*	**↓**
EMBV3_07237	NM_001075133	*PGRMC1*	**↓**
EMBV3_34603	NM_001110774	*C3orf57*	**↓**
EMBV3_01743	NM_001075980	*TM4SF1*	**↓**
EMBV3_41263	NM_001102503	*LOC520387*	**↓**
EMBV3_33131	XM_002699713	*MECP2*	**↓**
EMBV3_27274	NM_001103331	*C6orf120*	**↓**
EMBV3_21895	NM_001099036	*NCK2*	**↓**
EMBV3_15031	NM_001075156	*RPRD1A*	**↓**
EMBV3_30252	XM_002694236	*ZNF281*	**↓**
EMBV3_26127	NM_001081526	*PCMTD1*	**↓**
EMBV3_14309	NM_001102147	*FGFR1OP2*	**↓**
EMBV3_02754	XM_002686473	*THRAP3*	**↓**
EMBV3_25144	XM_002697779	*LOC100296226*	**↓**
EMBV3_14247	NM_001083654	*SLC35E3*	**↓**
EMBV3_34221	NM_001098050	*MAML2*	**↓**
EMBV3_35542	NM_001102223	*CDYL*	**↓**
EMBV3_11161	NM_001025319	*CYP51A1*	**↓**

Arrows ↑ and ↓ indicate up and down regulation in CVO compared to NVO blastocysts, respectively

**Table 3:** Differentially expressed 3′-UTR alternative variants between CVO and NVO groups

Probe ID	Target ID	Gene symbol	Expression patterns
EMBV3_15895	NM_001076288:853^956	*ZBTB8OS*	**↑**
EMBV3_11755	NM_001103313:118^183	*STAU2*	**↓**
EMBV3_29618	XM_002690797:448^620	*RTF1*	**↑**
EMBV3_23690	NM_001040581:162^233	*RPS21*	**↑**
EMBV3_04521	NM_001034434:91^236	*RPL30*	**↑**
EMBV3_11916	NM_001046138:573^772	*RHOC*	**↓**
EMBV3_04120	NM_001034266:602^718	*PSMB4*	**↑**
EMBV3_38466	NM_174749:398^529	*PRDX5*	**↑**
EMBV3_37565	NM_001025325:256^495	*PLAC8*	**↑**
EMBV3_30094	NM_001025325:256^380	*PLAC8*	**↑**
EMBV3_14878	NM_001034440:600^645	*PEBP4*	**↓**
EMBV3_12712	NM_001034384:651^728	*NOL7*	**↑**
EMBV3_00366	NM_001038133:297^732	*NIT2*	**↓**
EMBV3_13552	XM_002686892:558^606	*MYL7*	**↑**
EMBV3_35400	XM_002686892:374^478	*MYL7*	**↑**
EMBV3_16450	NM_175780:406^572	*MYL6*	**↑**
EMBV3_15619	NM_175780:458^502	*MYL6*	**↑**
EMBV3_22522	NM_175780:413^569	*MYL6*	**↑**
EMBV3_27866	NM_175780:273^550	*MYL6*	**↑**
EMBV3_27828	NM_175780:392^627	*MYL6*	**↑**
EMBV3_40962	NM_175780:313^568	*MYL6*	**↑**
EMBV3_35847	NM_001076018:227^306	*MTHFD1L*	**↑**
EMBV3_27125	NM_001046508:169^252	*MRPS18C*	**↑**
EMBV3_07488	NM_001075276:429^789	*MRPL55*	**↑**
EMBV3_08656	XM_002692789:248^375	*MGC148714*	**↑**
EMBV3_08932	XM_002685429:193^371	*LOC781039*	**↓**
EMBV3_04189	XM_002694113:286^391	*LOC616065*	↑
EMBV3_35024	XM_002685423:386^787	*LOC100337018*	**↓**
EMBV3_26700	XM_002685421:278^542	*LOC100336997*	**↓**
EMBV3_32203	XM_002690260:82^219	*IL20RA*	**↓**
EMBV3_13795	NM_001101264:282^515	*FERMT2*	**↑**
EMBV3_33655	NM_174217:747^992	*EZR*	**↑**
EMBV3_19809	NM_001075795:717^795	*EIF4E2*	**↑**
EMBV3_41916	NM_001015586:55^362	*DSTN*	**↑**
EMBV3_36018	NM_001033763:224^944	*DNAJB1*	**↓**
EMBV3_42215	NM_175807:98^187	*COX7A2*	**↑**
EMBV3_03749	NM_001077831:264^339	*COX6A1*	**↑**
EMBV3_34344	NM_001002891:521^562	*COX5A*	**↑**
EMBV3_08339	NM_001002891:400^444	*COX5A*	**↑**
EMBV3_28626	NM_001078036:814^858	*COBL*	**↑**
EMBV3_29989	NM_001001855:292^409	*BIRC5*	**↑**
EMBV3_28386	NM_001113719:163^210	*ATP5J2*	**↑**
EMBV3_25301	NM_174724:350^412	*ATP5H*	**↑**
EMBV3_07361	NM_176649:158^336	*ATP5G1*	**↑**

Arrows ↑ and ↓ indicate up and down regulation in CVO compared to NVO blastocysts, respectively

### Expression of gene cluster predicting the developmental capacity of in vivo derived bovine embryos

**Fig. 3:**
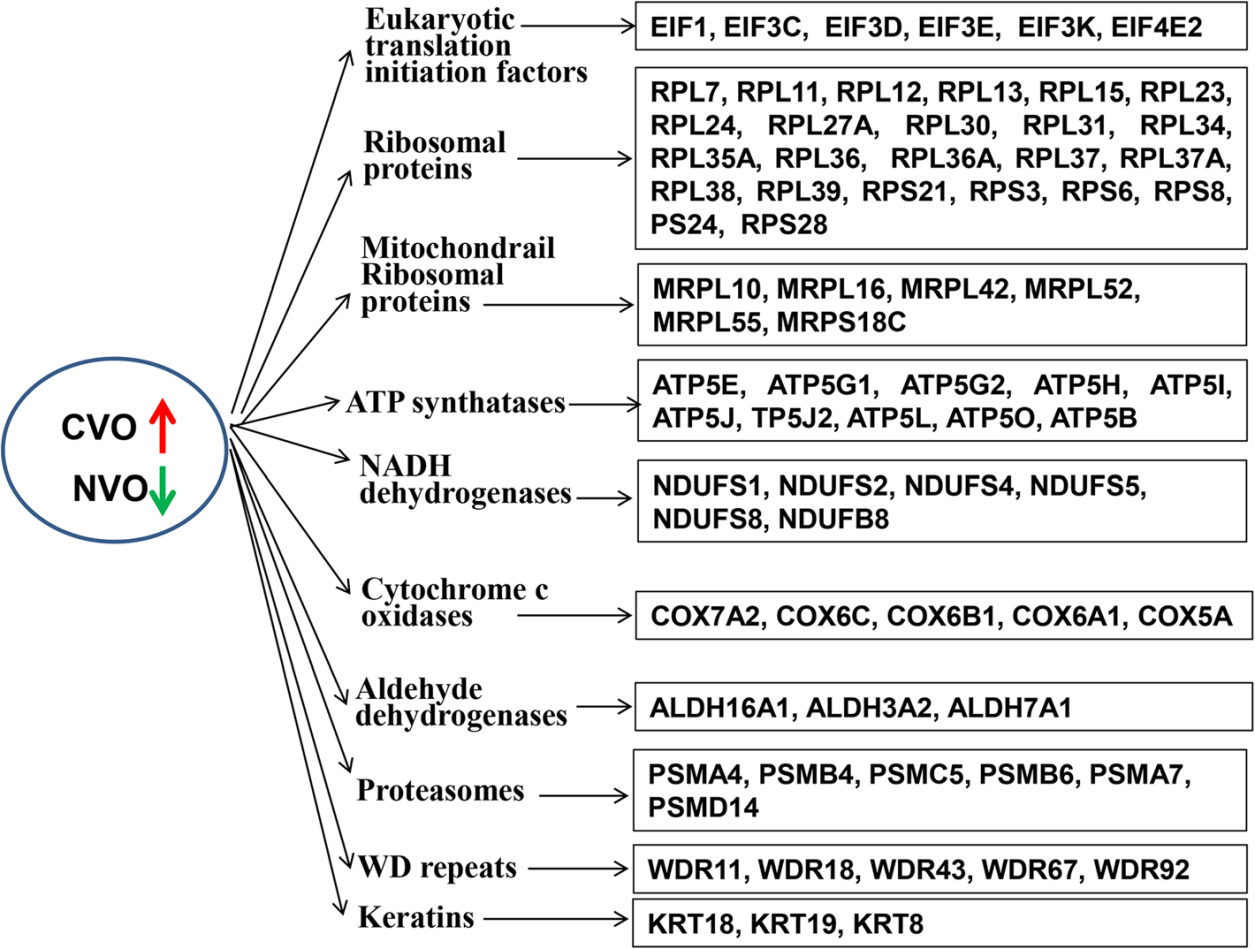
Gene clusters significantly enriched in in vivo derived competent embryos (CVO) compared to none competent (NVO) ones

In this study, we identified several gene cluster, each comprising of a group of genes potentially sharing a generalized function, exhibiting higher expression in CVO compared to NVO samples, that predict developmental capacity of in vivo derived bovine embryos. These gene cluster are mainly associated with mitochondrial functions and include ATP synthases (*ATP5E, −G1, −G2, −H, −I, −J, −J2, −L, −O*), eukaryotic translation initiation factors (*EIF1, −3C, EIF3D, −E, −K, EIF4E2),* ribosomal proteins (*RPL7, − 11, − 12, − 13. -15, − 23, − 24, − 27A, − 30, − 31, − 34, −35A, − 36, −37A, − 38, − 39, RPS3, − 6, − 8, − 21, − 24, − 28*), mitochondrial ribosomal proteins, NADH dehydrogenases (*NDUFS1, − 2, − 4, − 5, − 8, NDUFB8*), cytochrome c oxidases, aldehyde dehydrogenases, proteasomes, WD repeats and keratins (Fig. 3). Higher expression of these gene clusters specifically in the competent in vivo derived embryos (CVO) could indicate the upregulation of global protein translation turnover and ATP generating pathways.

### Molecular signatures predicting the developmental capacity of in vitro derived bovine embryos

**Fig. 4:**
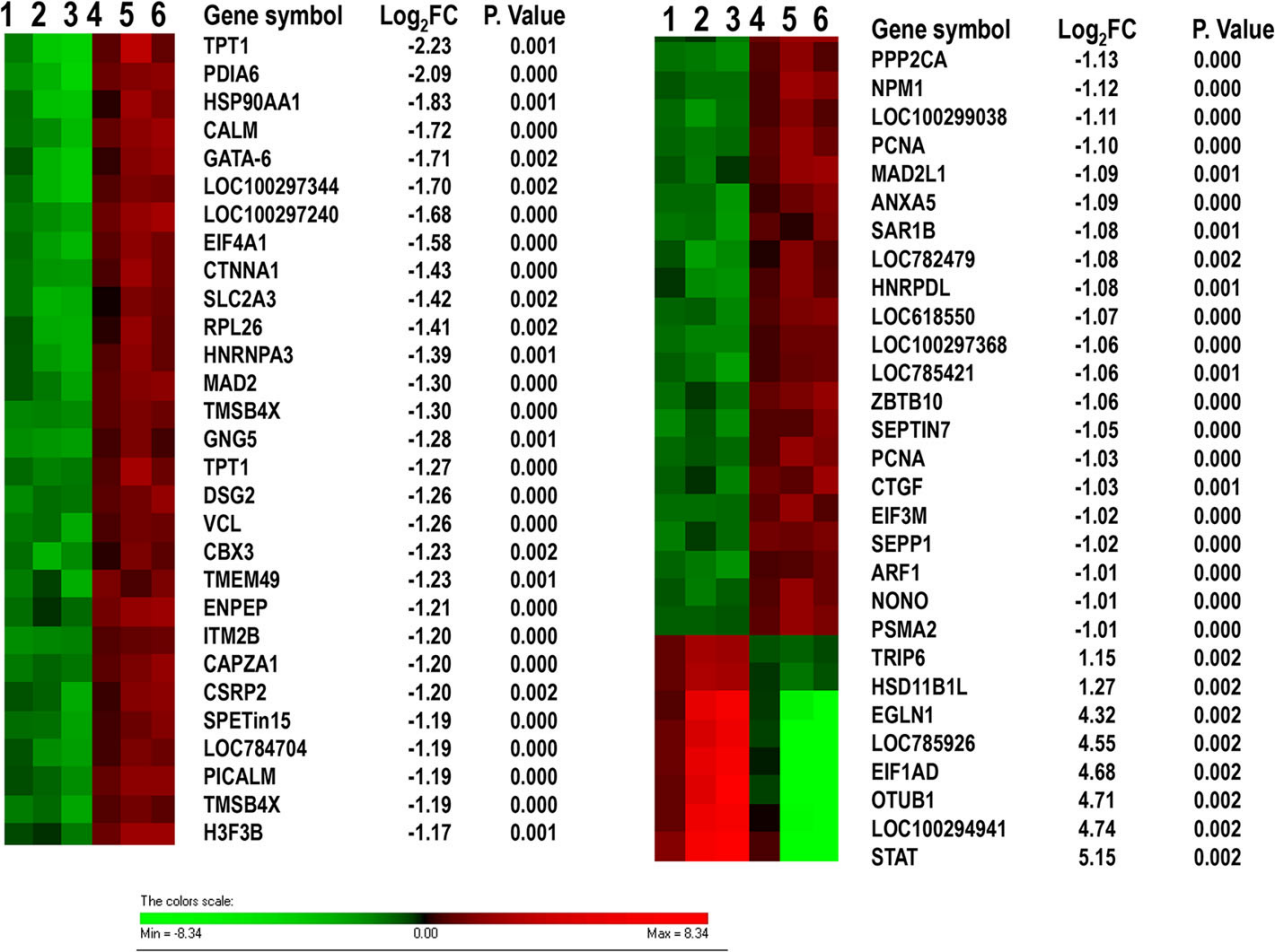
Molecular signature associated with developmental capacity of in vitro derived embryos. The heatmap indicates the expression patterns of the top 58 differentially expressed genes between CVT and NVT. Numbers 1, 2, 3 indicate three biological replicates hybridization whereas 1 μg of labelled Cy-5 labelled CVT samples were hybridized with 1 μg of Cy-3 labelled NVT samples. Numbers 4, 5, 6 indicate dye-swaps in which 1 μg of labelled Cy-3 labelled CVT samples were hybridized with 1 μg of Cy-5 labelled NVT samples

The gene expression analysis in the competent in vitro derived embryos (CVT) and non-competent in vitro derived embryos (NVT) at blastocyst stage showed a differential expression of 218 gene transcripts (226 probes) (Table 4, Supplemental Table S2). Of these, the expression of 194 genes was increased while expression of 24 genes was decreased in CVT compared to NVT samples. Among the DEGs, *TPT1*, *PDIA6*, *HSP90AA1* and *CALM* were among the top upregulated genes demonstrating differential expression by 3.5–4.5 folds (*p* < 0.05) whereas *STAT1*, *OTUB1*, *EIF1AD* and *EGLN1* were among the top downregulated genes (23–35 folds) in CVT compared to NVT group (Fig. 4). Moreover, about 1.8 and 8.4% of all DEGs represented splice variants (Table 5) and alternative 3′-UTR events (Table 6), respectively. However, the total number of DEGs between CVT vs. NVT was 3.2 times lower compared to the total number of DEGs obtained in CVO vs. NVO groups.

**Table 4:** Preferentially expressed probes in CVT and NVT groups

	Enriched in CVT	Enriched in NVT	Total
Constitutive (not discriminating variants)	15	157	172
Novel gene; evidence: embryonic ESTs (NTR)	3	28	31
Alternative 3′UTR events (genes)	1	18	19
Indel type splice variants	4	0	4
Total differentially expressed probes	23	203	226

**Table 5:** Differentially expressed gene variants between CVT and NVT groups

Probe ID	Target ID	Gene symbol	Expression patterns
EMBV3_09669	NM_001034719:575^658	*OTUB1*	↑
EMBV3_11755	NM_001103313:118^183	*STAU2*	↑
EMBV3_12635	NM_001035469:214^341	*TRIP6*	↑
EMBV3_19031	NM_206969:327^438	*HSD11B1L*	↑

Arrow (↑) indicates upregulation genes in the CVT group compared to the NVT ones

**Table 6:** Differentially expressed 3′-UTR alternative variants between CVT and NVT groups

Probe ID	Gene symbol	Description	Expression patterns
EMBV3_30252	*ZNF281*	Zinc finger protein 281	**↓**
EMBV3_40010	*TMEM170A*	TMEM170 transmembrane protein 170A	**↓**
EMBV3_03965	*SYPL1*	Synaptophysin-like 1	**↓**
EMBV3_08091	*SYAP1*	Synapse associated protein 1, SAP47 homolog (Drosophila)	**↓**
EMBV3_42313	*SNX4*	Sorting nexin 4	**↓**
EMBV3_14247	*SLC35E3*	Solute carrier family 35, member E3	**↓**
EMBV3_05094	*SEPTIN7*	Septin 7	**↓**
EMBV3_13169	*RELL1*	RELT-like 1	**↓**
EMBV3_21053	*PPP1CC*	Protein phosphatase 1, catalytic subunit, gamma isoform	**↓**
EMBV3_25376	*KCTD8*	Potassium channel tetramerisation domain containing 8	**↓**
EMBV3_30092	*PLEKHF2*	Pleckstrin homology domain containing, family F (with FYVE domain) member 2	**↓**
EMBV3_33562	*PHF3*	PHD finger protein 3	**↓**
EMBV3_16278	*NCOA4*	Nuclear receptor coactivator 4	**↓**
EMBV3_07677	*M6PR*	CDMPR MGC140730 mannose-6-phosphate receptor (cation dependent)	**↓**
EMBV3_41263	*LOC520387*	KIAA0528-like	**↓**
EMBV3_26821	*DSG2*	Desmoglein 2	**↓**
EMBV3_11161	*CYP51A1*	Cytochrome P450, family 51, subfamily A, polypeptide 1	**↓**
EMBV3_35542	*CDYL*	Chromodomain protein, Y-like	**↓**
EMBV3_16966	*CANX*	Calnexin	**↓**

Arrow (↓) indicates the downregulation genes in CVT compared to NVT blastocysts

### Molecular functions and pathways predicting developmental capacity

**Fig. 5:**
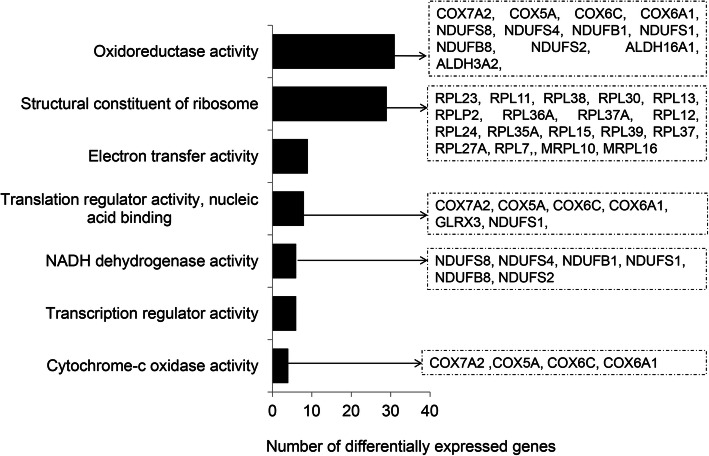
Molecular functions significantly enriched by differentially expressed genes in competent in vivo derived embryos (CVO vs. NVO). Lists of genes on the right indicate differentially expressed genes involved within these distinct molecular functions

**Fig. 6:**
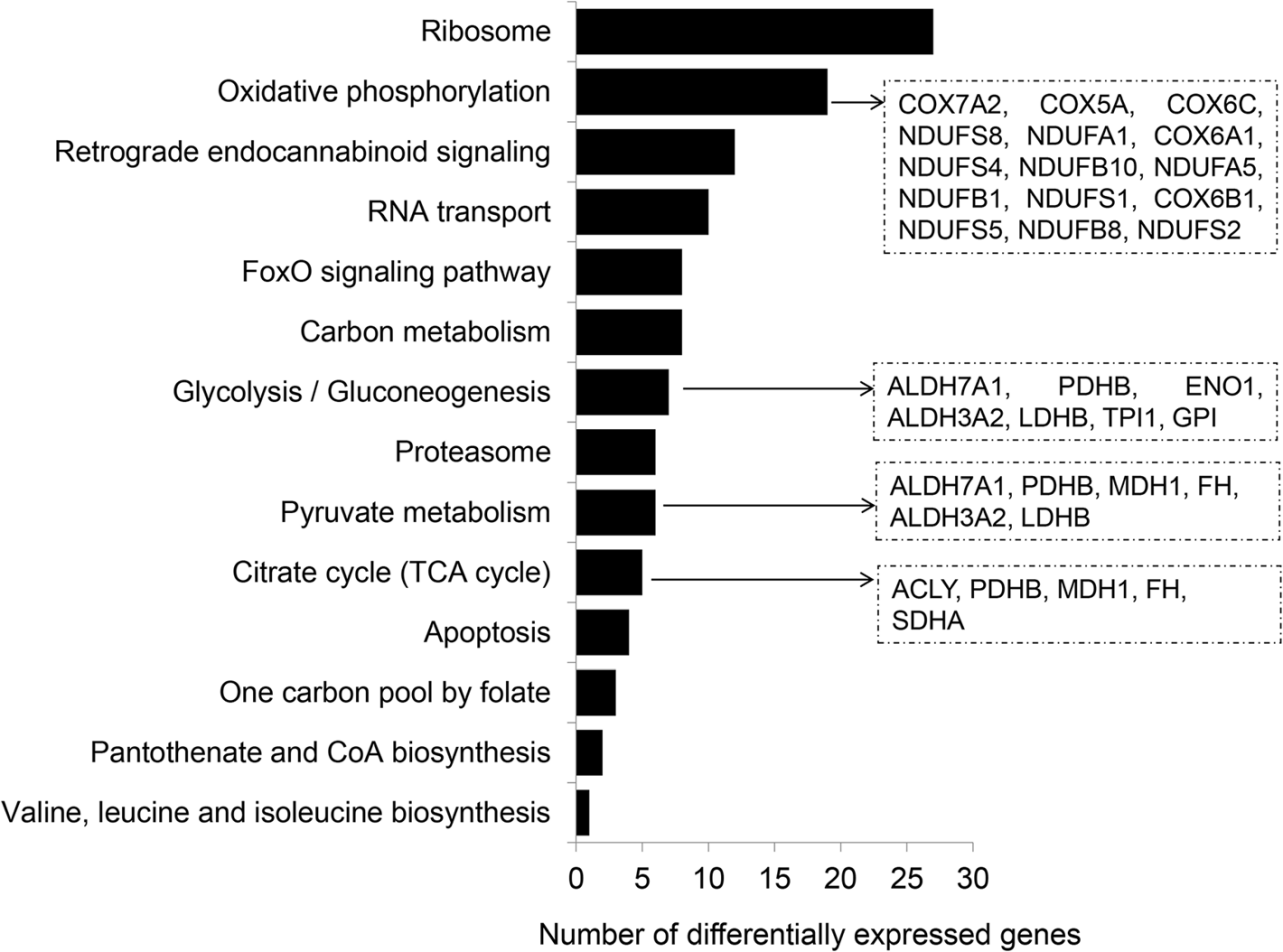
Molecular pathways significantly enriched by differentially expressed genes in competent in vivo derived embryos (CVO vs. NVO). Lists of genes on the right indicate differentially expressed genes involved within these distinct molecular pathways

**Fig. 7:**
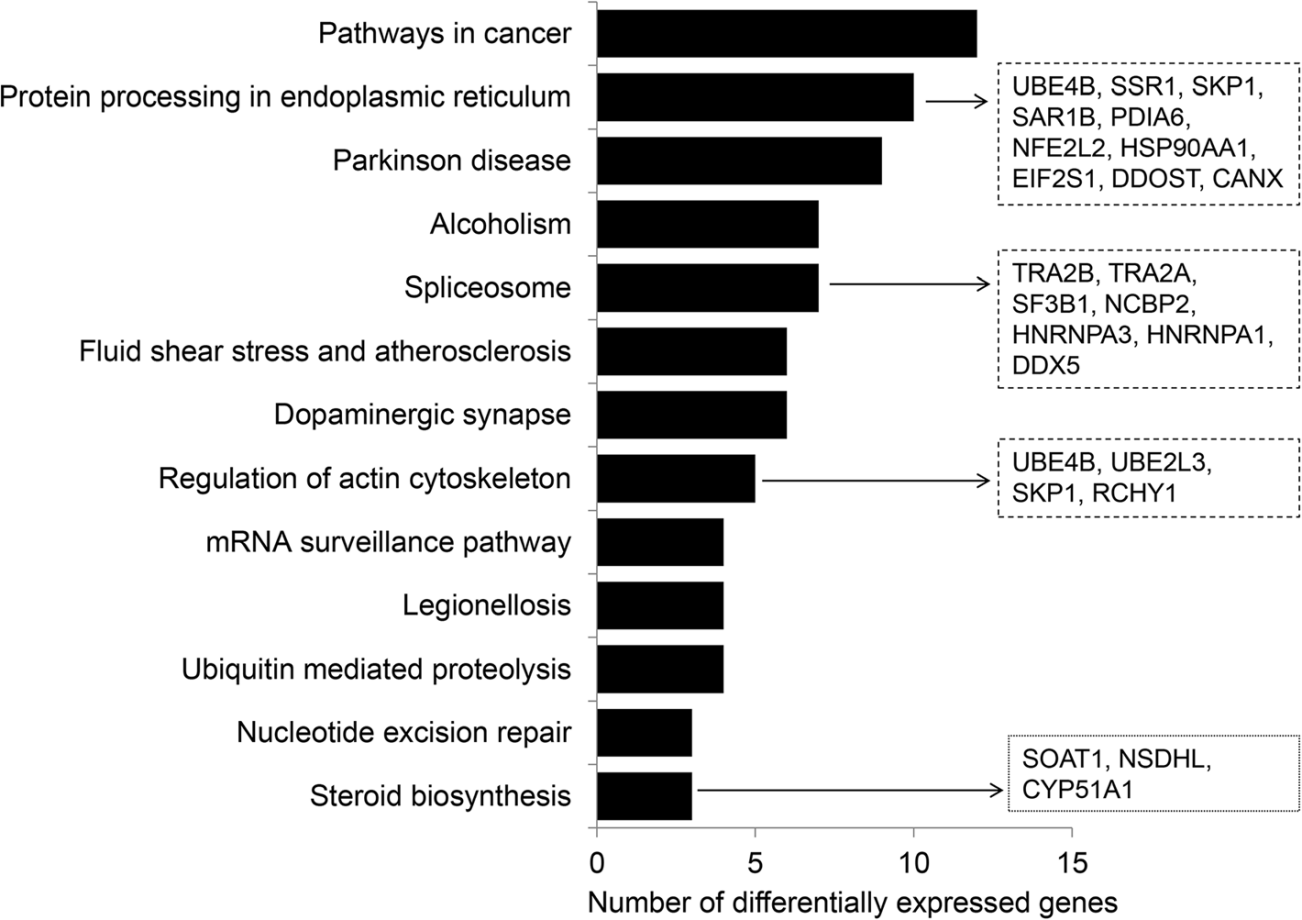
Molecular functions significantly enriched by differentially expressed genes specifically in competent in vitro derived embryos (CVT vs. NVT). Lists of genes on the right indicate differentially expressed genes involved within these distinct molecular functions

To unravel relevant molecular functions and pathways in competent in vivo derived embryos, we performed gene ontological enrichment analysis of preferentially expressed genes in CVO and NVO groups using the g: Profiler bionformatic tool. Accordingly, those DEGs were found to be mainly involved in ATP production related molecular functions (oxireducatase activity, electron transfer activity, cytochrome c oxidase activity and NADH dehydrogenase activity) (Fig. 5) and KEGG pathways associated with energy metabolism and transformation (glycolysis/glycogenesis, citrate acid cycle, pyruvate metabolism and oxidative phosphorylation), foxo signaling and proteasome) (Fig. 6). Likewise, we have also investigated the relevant molecular functions and pathways in bovine in vitro derived developmentally competent embryos. Gene ontology enrichment analysis showed that preferentially expressed genes in CVT and NVT were found to be involved in translation initiation factor activity, nucleic acid binding, protein binding, actin filament binding and actin filament binding molecular functions (Supplemental Table S3). Moreover, those DEGs were found to be involved in 13 KEGG pathways including protein processing in endoplasmic reticulum, spliceasome, ubiquitone mediated proteolysis and steroid biosynthesis (Fig. 7).

### Expression of genes predictive for the developmental capacity in ICM and TE cells

**Fig. 8:**
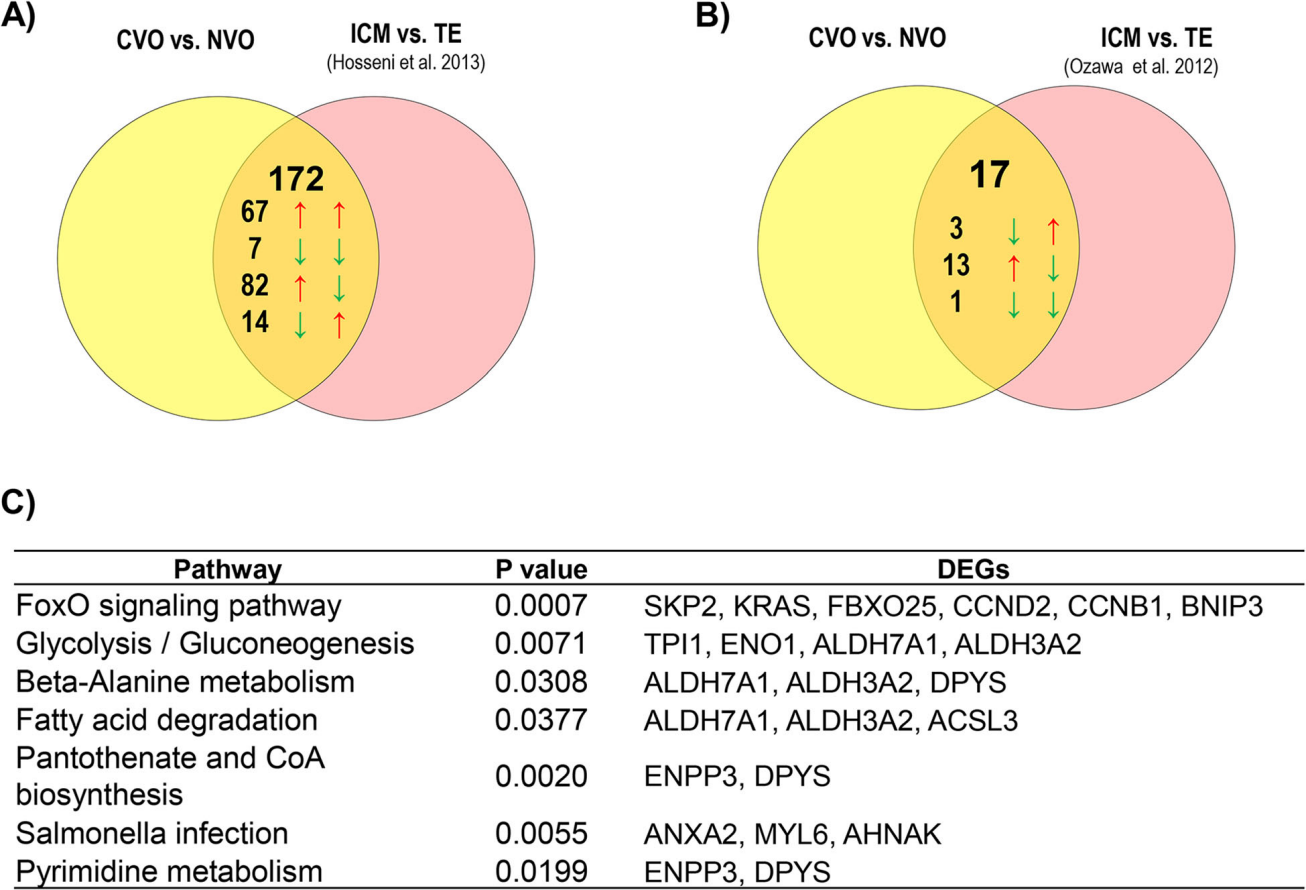
Meta-analysis of genes predictive for developmental capacity of in vivo derived embryos and differential expressed between ICM and TE cells. **a** Venn diagram depicting genes differentially expressed in CVO vs. NVO as well as in ICM vs. TE cells of in vivo derived embryos. The arrows on the left hand indicate expression trend of genes in CVO compared to NVO blastocysts whereas the arrows on the right side indicate expression trend of these genes in ICM relative to TE cells of in vivo derived embryos. **b** Venn diagram depicting genes differentially expressed in CVO vs. NVO as well as in ICM vs. TE cells of in vitro derived embryos. The arrows on the left indicate expression trend of genes in CVO compared to NVO blastocysts whereas the arrows on the right side indicate expression trend of these genes in ICM relative to TE cells of in vitro derived embryos. **c** Molecular pathways enriched by genes differentially expressed both in CVO vs. NVO blastocysts and in ICM vs. TE cells

Mammalian embryos’ ability to induce a pregnancy is believed to be dependent on proper specialization the totipotent embryonic cells into pluripotent inner cell mass (ICM) and trophectoderm (TE) cells. This processes is in turn governed by preferential expression of typical molecular signatures in ICM and TE cells [41]. With respect to this, we conducted a meta-analysis by comparing the DEGs identified in embryos of CVO vs. NVO groups with the gene expression outline of ICM and TE cells of in vivo [42] and in vitro [43] derived bovine blastocysts. Interesting, 172 DEGs reported to be differentially expressed between ICM and TE cells of in vivo derived bovine blastocysts [42] and 17 DEGs reported to be by differentially expressed between ICM and TE cells of in vitro derived blastocysts [43] were also found to be differentially expressed between CVO and NVO blastocysts in the present study (Fig. 8a & b, Supplemental Table S4). Of these, a total of 67 genes including the ribosomal proteins (*RPS8, RPS21, RPLP2, RPL39, RPL38, RPL36A, RPL31, RPL30, RPL24, RPL15, RPL13* and *RPL11*) which were upregulated in CVO compared to NVO embryos were also upregulated in ICM compared to TE cells of in vivo derived blastocysts [42]. On the other hand, a total of 82 genes including *KRT19, KRT8, CTSZ, KRT18,* and *MYL6* were downregulated in CVO vs. NVO groups but upregulated in TE vs. ICM cells. Likewise, 14 genes which were downregulated in CVO vs. NVO including *ZNF281*, *NANOG*, *FOSL1* and *DPYS* were upregulated in ICM compared to TE cells whereas 7 genes (*RPRD1A, RHOC, PLXDC2, PGRMC1, CYP51A1, C6orf120* and *ACLY*) which were downregulated in CVO vs. NVO were also downregulated in ICM vs. TE cells. Functional annotation showed that some of these genes were enriched in distinct pathways namely Foxo signalling pathway, glycolysis/gluconeogenesis, beta-Alanine metabolism, pantothenate and CoA biosynthesis, pyrimidine metabolism and fatty acid degradation pathways (Fig. 8c).

**Fig. 9:**
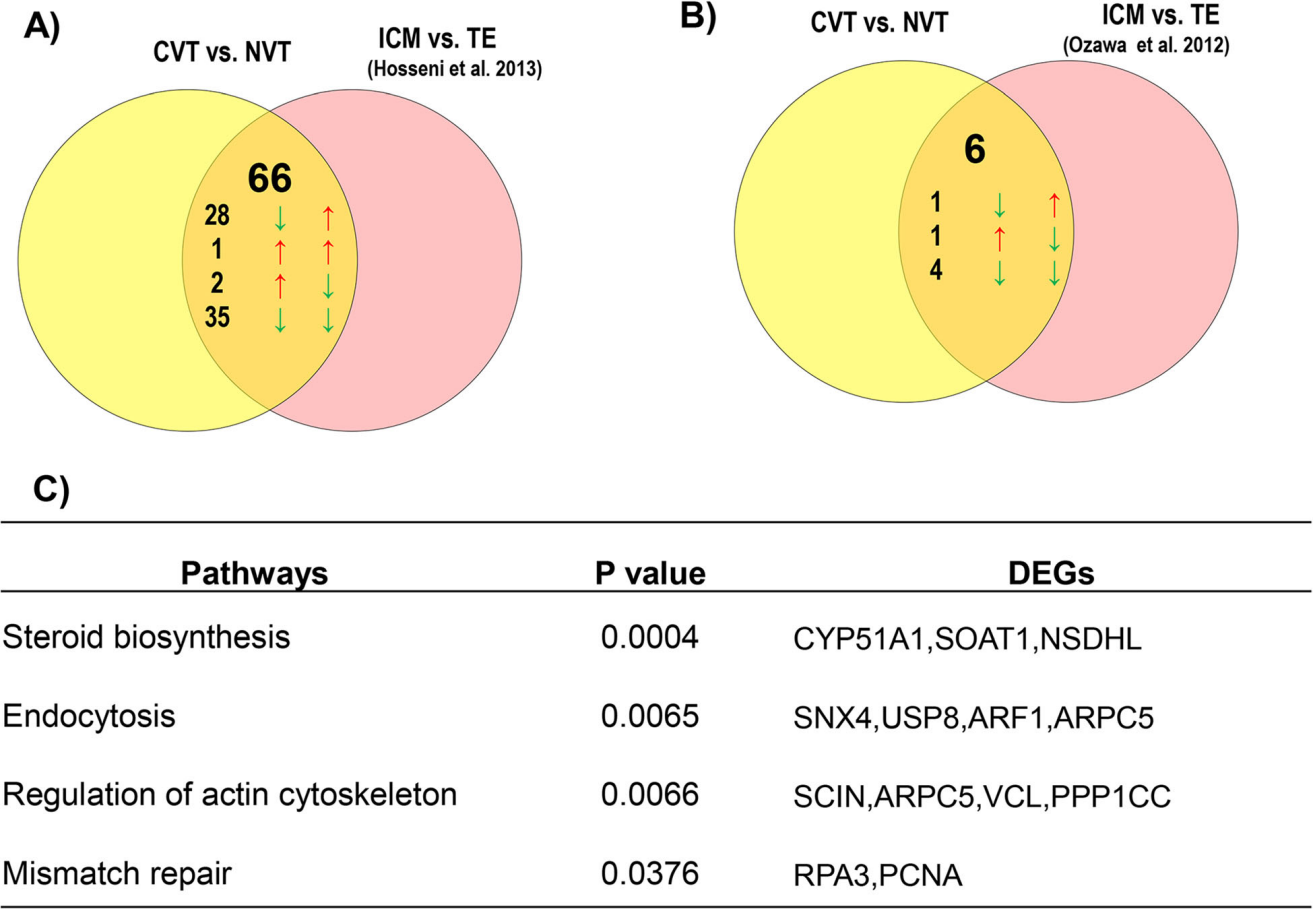
Meta-analysis of genes predictive for developmental capacity of in vitro derived embryos and differential expressed between ICM and TE cells. **a** Venn diagram depicting genes differentially expressed in CVT vs. NVT as well as in ICM vs. TE cells of in vivo derived embryos. The arrows on the left indicate expression trend of genes in CVT compared to NVT blastocysts whereas the arrows on the right side indicate expression trend of these genes in ICM relative to TE cells of in vivo derived embryos. **b** Venn diagram depicting genes differentially expressed in CVT vs. NVT as well as in ICM vs. TE cells of in vitro derived embryos. The arrows on the left indicate expression trend of genes in CVT compared to NVT blastocysts whereas the arrows on the right side indicate expression trend of these genes in ICM relative to TE cells of in vitro derived embryos. **c** Molecular pathways enriched by genes differentially expressed both in CVT vs. NVT blastocysts and as in ICM vs. TE cells

The DEGs identified between competent and non-competent in vitro derived blastocysts (CVT vs. NVT) were also merged to studies of Hosseini et al. [42] and Ozawa et al. [43] . This analysis has shown that a total of 66 and 6 annotated DEGs previously reported to be differentially expressed in ICM vs. TE cells of in vivo [42] and in vitro derived blastocysts [43] were also differentially expressed between CVT and NVT blastocysts in the present study (Fig. 9a & b, supplemental Table S5). Of those, 28 genes including *PPP1CC*, *ZNF281*, *H3F3B* and *H2AFZ* reported previously to be enriched in ICM cells [42] were downregulated in CVT compared to NVT embryos whereas 35 genes obtained to be downregulated in CVT vs. NVT embryos in the present study including *FERMT2*, *SLC16A1*, *SNX4*, *TXN* and *PDIA6* were reported to be enriched in TE cells (Supplemental Table S5). Bioinformatic analysis showed that these DEGs were involved in steroid biosynthesis, endocytosis, regulation of actin cytoskeleton, mismatch repair (Fig. 9c).

### Molecular signatures reflecting environmental conditions in competent bovine embryos

`Understanding the differences in the gene expression outline between developmentally competent in vitro and in vivo originated blastocyst is suggested to be useful to identify gene expression signatures associated with pathophysiological postnatal consequences caused by the environment during in vitro embryo production. Therefore, here we investigated differences in terms of gene expression signatures specifically affected by developmental environment in competent in vitro derived blastocysts (CVT) vs. competent vivo derived ones (CVO). Including novel transcripts (NTRs), alternative 3′-UTR events, Indel-type splice variants and pseudogenes, a total of 1066 probes associated with 937 transcripts were differentially expressed between competent in vivo and in vitro derived embryo groups indicating differences although both groups had resulted in establishment of pregnancy. The expression trend of 83.2% of all DEGs including *RPS27A, RPS21, RPS13, EEF1A1* and *CYCS* was reduced whereas the expression of 16.7% of all DEGs including *SEMA6C*, *TPRF*, *NFATC4* and SMARCA2 was increased in CVT vs. CVO samples (Supplemental Fig. 1, Supplemental Table S6). In addition, a total of 50 DEGs including *RPLP0*, *COX5A*, *ATP5J2* and *ATP5C1* represented indel type splice variants (Table 7) whereas a total of 83 differentially expressed probes including those associated with *TPM4*, *SLC31A1*, *INA*, *CS*, *TP53INP1*, *NCOA1*, *ATF1* and *SLC1A3* genes represented alternative 3′-UTR variants (Supplemental Table S7).

**Table 7:** Differentially expressed probes between CVT and CVO groups

	Enriched in CVT	Enriched in CVO	Total
Constitutive (not discriminating variants)	124	562	686
Novel gene; evidence: embryonic ESTs (NTR)	54	193	247
Alternative 3′UTR events (genes)	15	68	83
Indel type splice variants	18	32	50
Pseudogenes	0	28	28
Total differentially expressed probes	211	855	1066

### Expression of gene cluster reflecting the environmental conditions in competent bovine embryos

**Fig. 10:**
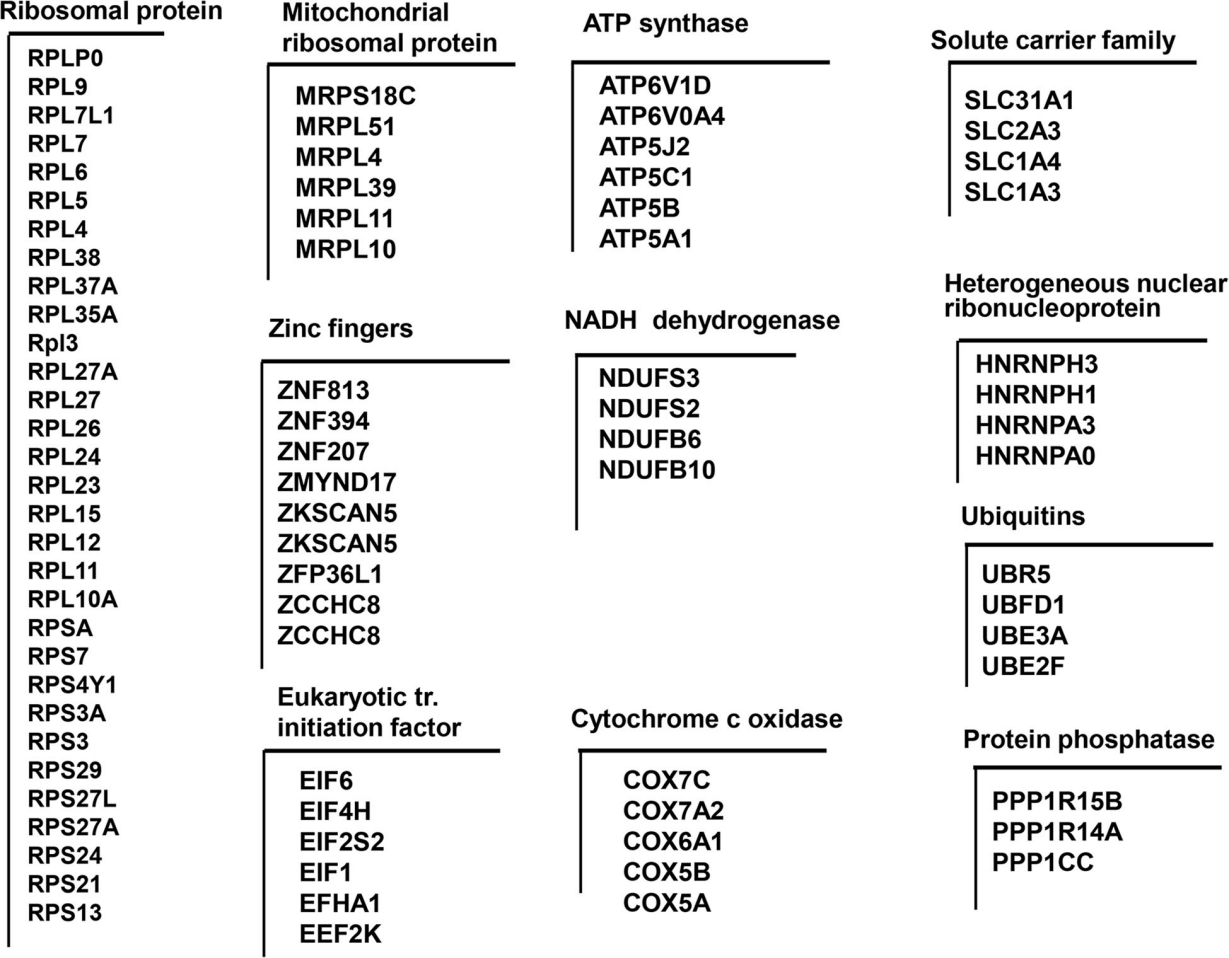
Arrays of gene cluster reflecting environmental conditions in competent embryos (CVT vs. CVO)

In addition to characterize individual differential expressed genes as novel transcripts (NTR), alternative 3′-UTR events, indel type splice variants or pseudogenes, we have also identified some gene cluster bearing similar expression patterns in one sample group relative to another one which is a step forward for selecting promising candidate genes associated with the trait of interest. Accordingly, we have investigated the expression patterns of genes which share similar characteristics and biochemical functions. Thus, a detailed analysis has shown that several cluster of genes including ribosomal proteins (*n* = 36), zinc fingers (*n* = 9), solute carriers (*n* = 7), mitochondrial ribosomal proteins (n = 9), eukaryotic translation initiation factor (*n* = 5), nuclear ribonucleoprotein (*n* = 6) and NADH dehydrogenase (*n* = 4) were reduced in the CVT group compared to the CVO ones (Fig. 10). Of the 36 differentially expressed ribosomal proteins, the expression of ribosomal proteins including *RPS27A, RPS21, RPS13, RPL12, RPL27, RPL7, RPS24, RPS3, RPS29* and *RPS21* exhibited 4–8 fold change reduction in CVT.

### Molecular functions and pathways reflecting environmental conditions of competent bovine embryos

**Fig. 11:**
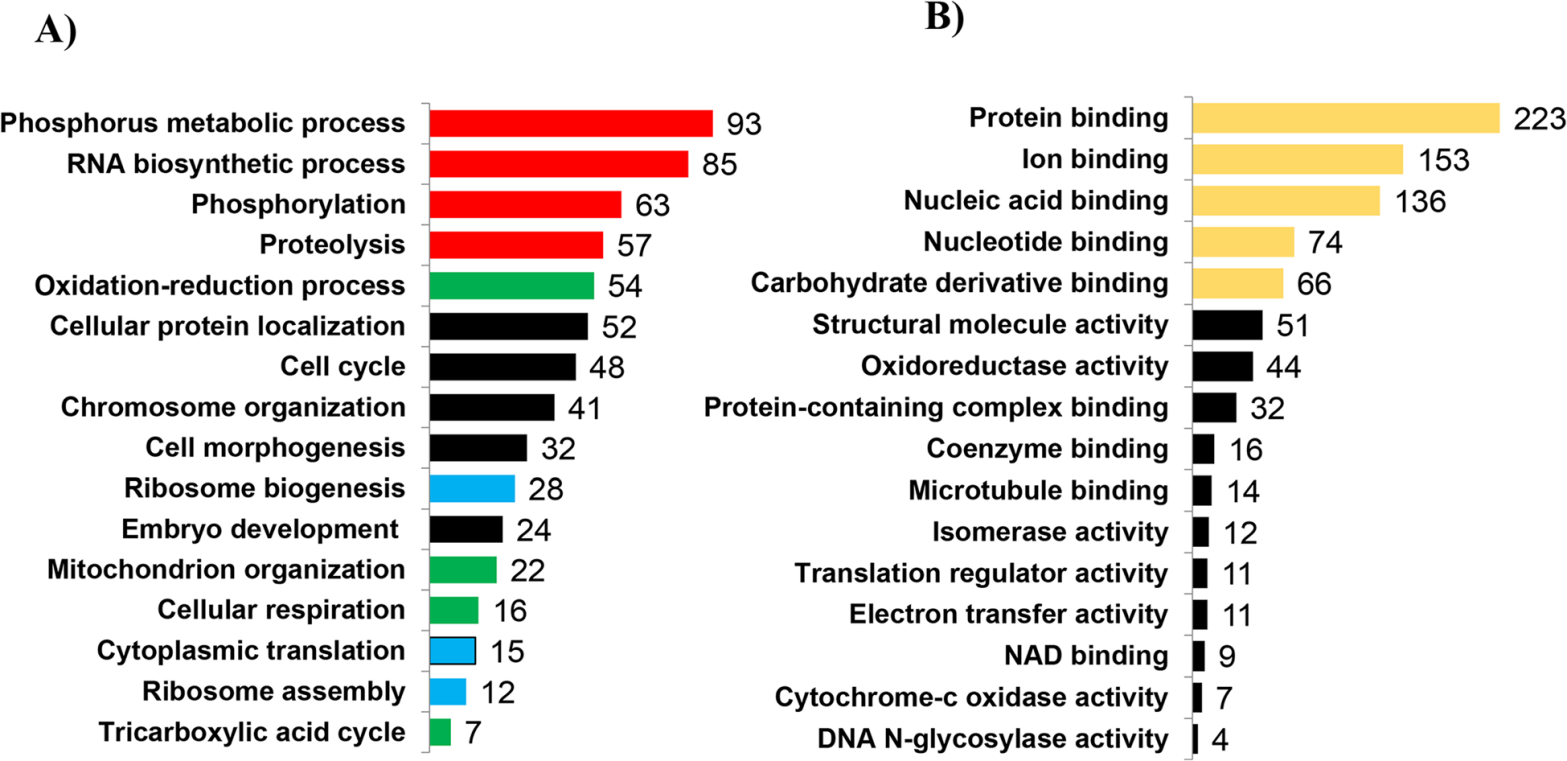
Biological processes (**a**) and molecular functions (**b**) significantly enriched by differentially expressed genes specifically modulated by the environmental conditions in competent embryos (CVT vs. CVO). Red, green, black and blue bars in Fig. A indicate functions associated with metabolism, energy production, cell cycle related activities and protein synthesis, respectively whereas yellow and black bars in Fig. B indicate binding and enzymatic activities respectively

Gene ontology enrichment analysis showed that those differentially expressed genes between CVT and CVO samples were found to be involved in biological processes associated with metabolism, ATP production, cell cycle related activities, and protein synthesis (Fig. 11a, supplemental Table S8). In addition, those DEGs were also found to be involved in molecular functions including binding activity, oxireductase activity, cytocrom-c reductase activity, electron transfer activity and catalytic activities (Fig. 11b, supplemental Table S9). In line with that, these DEGs were involved in distinct molecular pathways including translation, energy metabolism, transport and catabolism, cell growth and death, folding, sorting and degradation, carbohydrate metabolism, replication, and repair (Supplemental Fig. 2).

### Molecular signatures reflecting environmental conditions exclusively in competent bovine embryos

**Fig. 12:**
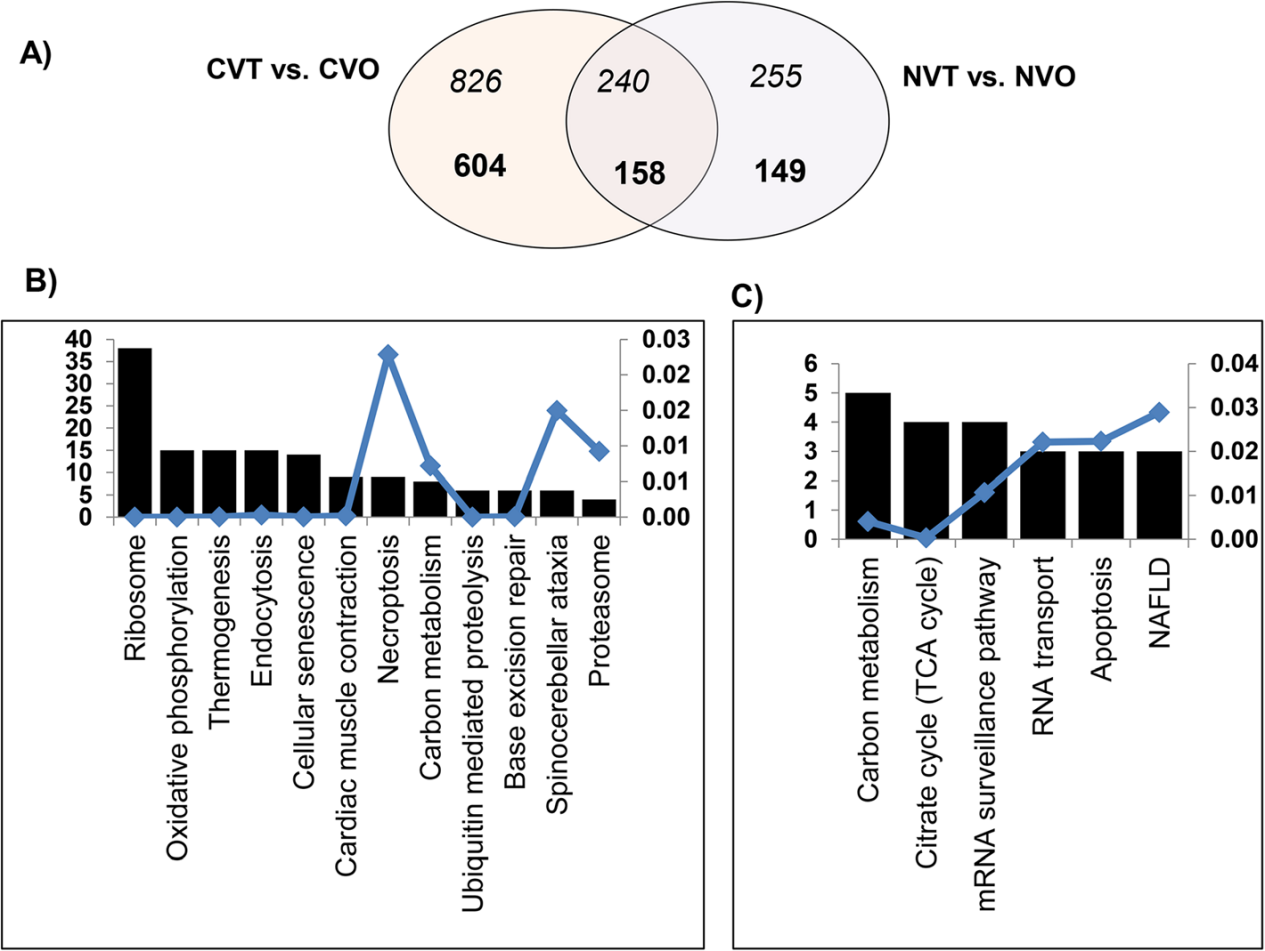
Summary of (**a**) differential expressed genes modulated by environmental conditions in competent embryos (left, CVT vs. CVO) and non-competent embryos (right, NVT vs. NVO). Venn diagram reports also number of genes differential expressed in common (center). Total numbers of differential expressed probes for each section are presented in italic whereas annotated ones are indicated in bold. **b** Molecular pathways significantly enriched by genes affected by environmental conditions exclusively in competent embryos (CVT vs. CVO). **c** Molecular pathways significantly enriched by genes affected by environmental conditions both in competent and non-competent embryos. Black bars indicate numbers of differentially expressed genes and blue dots indicate adjusted *p* values. NAFLD: Non-alcoholic fatty liver disease

Identification of genes being exclusively differentially expressed between competent in vivo derived and competent in vitro derived embryos (CVT vs. CVO) and between non-competent in vivo derived embryos and non-competent in vitro derived embryos (NVT vs. NVO) was done to identify genes which were specifically affected by the culture environment without being conflictive with further development if aberrantly expressed. The result from this analysis indicated as much as four times more exclusively differentially expressed annotated genes between CVT vs. CVO compared to those detected when comparing NVT vs. NVO groups (Fig. 12a). Exclusively differentially expressed genes in CVT vs. CVO include several gene clusters such as ribosomal proteins, zinc fingers, solute carriers, mitochondrial ribosomal proteins, eukaryotic translation initiation factors, nuclear ribonucleoprotein and NADH dehydrogenase. In agreement, genes exclusively differentially expressed in the CVT vs. CVO groups were found to be involved in several pathways including oxidative phosphorylation, ubiquitin mediated proteolysis, cellular senescence, and proteasome pathways (Fig. 12b).

### Molecular signatures reflecting environmental conditions in non-competent embryos

The transcriptome profile comparison between non-competent in vitro (NVT) and non-competent in vivo blastocysts identified a total of 495 differential expressed probes encompassing 307 annotated and 188 novel transcripts (NTRs). Of these, 85.8% were down regulated in NVT group compared to NVO ones. Since, both NVT and NVO were confirmed not to end up in an initial pregnancy, different expressed genes between these two blastocysts groups is purely due to contrasting culture conditions*.* Among those, a total of 158 differentially expressed annotated genes were found to be common in the CVT vs. CVO comparison and. These genes were found to be preferentially involved in 6 KEGG pathways including citrate cycle (TCA cycle), carbon metabolism and apoptosis pathways (Fig. 12c).

### Expression of genes reflective for the developmental environment in ICM and TE

**Fig. 13:**
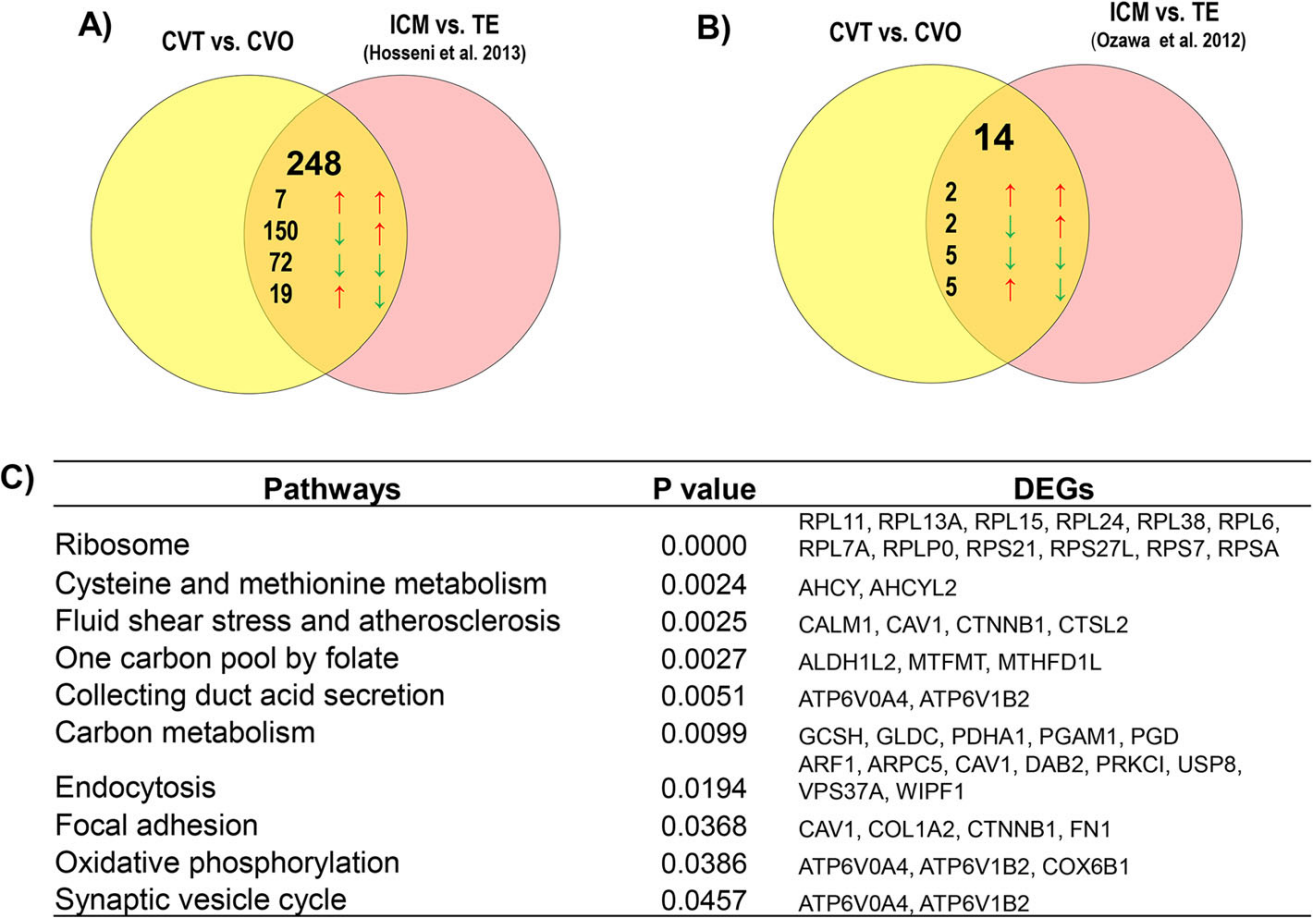
Meta-analysis of genes reflective for developmental environment and differential expressed between ICM and TE cells. **a** Venn diagram depicting genes differentially expressed in CVT vs. CVO as well as in ICM vs. TE cells of in vivo derived embryos. The arrows on the left hand indicate expression trend of genes in CVT compared to CVO blastocysts whereas the arrows on the right side indicate expression trend of these genes in ICM relative to TE cells of in vivo derived embryos. **b** Venn diagram depicting genes differentially expressed in CVT vs. CVO as well as in ICM vs. TE cells of in vitro derived embryos. The arrows on the left hand indicate expression trend of genes in CVT compared to CVO blastocysts whereas the arrows on the right side indicate expression trend of these genes in ICM relative to TE cells of in vitro derived embryos. **c** Molecular pathways enriched by genes differentially expressed both in CVT vs. CVO blastocysts as well as in ICM vs. TE cells

We also superimposed the genes preferentially expressed between the competent vivo and in vitro blastocysts (CVT vs. CVO) to those genes previously reported to be differentially expressed for ICM vs. TE cell of in vivo [42] and in vitro derived bovine blastocysts [43], respectively as indicated above. Accordingly, a total of 248 and 14 DEGs detected in CVT vs. CVO were identified as differentially expressed between ICM and TE cells of in vivo [42] and in vitro [43] derived bovine blastocysts, respectively (Fig. 13a & b). Of these, 157 genes have been shown to be enriched in ICM cells, whereas 91 DEGs have been reported to be enriched in TE cells of in vivo derived blastocysts, respectively (Fig. 13a, Supplemental Table S10). These genes are enriched in ribosome, cysteine, and methionine metabolism, one carbon pool by folate focal adhesion and oxidative phosphorylation pathways (Fig. 13c).

## Discussion

Mammalian preimplantation embryogenesis involves entire developmental stages from the formation of the diploid zygote up to formation of the blastocyst. After the blastocysts stage, the trophectoderm of the developing embryo gives rise to extra embryonic tissues and the epiblast differentiates into somatic lineages and the germline [44]. It is known that during the first successive cleavage stages, the embryo depends on the maternal stores accumulated during oocyte growth. After activation of its own genome, the embryo depends on transcripts and proteins that are de novo synthesized by the embryonic cells which begins at 4–8 cell stage in humans [45, 46], at 2-cell in mouse [47] and 8–16 cell stages in bovine [48]. For instance, a progressive increase in gene expression takes place during the early cleavage stages and morulae formation of mouse embryos [49]. This suggests that spatial and temporal dynamic expression of developmentally related genes and successful transitions from maternal to embryonic transcription is critical for successful embryonic development. Thus, identification of those transcripts that could favour embryonic development and sustain pregnancy could be an important step for developing genetic markers for selecting developmentally competent embryos for increasing pregnancy rates after embryo transfer. Not only the intrinsic developmental capacity, but also the developmental environment has been reported to modulate an embryo’s gene expression outline. Thus, interpretation of an embryo’s transcriptome in relation to its developmental capacity, while neglecting environmental conditions might lead to a wrong conclusion. Similarly, interpretation of embryos transcriptome profiles modulated by environmental conditions, while neglecting an embryo’s intrinsic developmental capacity might also lead to false results. Therefore, here we aimed to unravel specific molecular signatures of the bovine embryo and their predictive power with respect to its developmental capacity, contrasting environments and their interaction.

### Molecular signatures reflecting embryos’ developmental environment

Although in vitro produced embryos have some potential to develop to term, numerous reports elsewhere have reported prenatal and postnatal consequences in pregnancies established after transfer of in vitro derived embryos. Indeed, negative effects of the none-physiological in vitro culture environment on an embryo’s developmental potential could be a consequence of alterations of the gene expression outline or because of epigenetic modifications. In this respect, several candidate genes and large scale transcriptome profile analysis approaches [18–24] and DNA methylation studies [25–30] have proved profound effects of culture conditions on gene expression patterns and epigenetic profiles in the resultant blastocysts. However, in all these studies, no relevance was given to the developmental capacity of the individual embryo taken for (epi-)genome expression analysis. It is generally accepted, that lower proportions of in vitro derived embryos end up in calf delivery after transfer to recipients (about 15–41%) compared to in vivo derived counterparts (about 46%), as reviewed elsewhere [50]. Thus, to avoid incorrectly interpreting an embryo’s environmental condition-induced transcriptome profile changes, it is necessary to consider only embryos of comparable developmental capacity for comparative gene expression analysis. On the contrary, comparative gene expression profiles and epigenetic studies in the in vitro and in vivo originated embryos have usually been performed without determining the individual developmental competence of the embryos. Consequently, the results derived from these studies are a mixture of both competent and non-competent embryos. Conversely, in the present study, we investigated the transcriptome profile differences between competent in vivo (CVT) and in vitro (CVO) derived embryos as well as non-competent in vivo (NVT) and in vitro (NVO) derived embryos separately. Interestingly, the total number of differentially expressed genes in those comparisons revealed that an embryo’s transcriptome profile specifically modulated by the developmental environment seems to be more divergent in competent embryos than in non-competent ones (Fig. 12). In addition, several clusters of genes including those involved in ribosomal protein synthesis, ATP generating activities, and those involved in oxidative phosphorylation (Fig. 10) and several gene variants (Supplemental Table S11) were exclusively downregulated in blastocysts cultured in vitro. However, the main question that could be raised with respect to this issue is, why competent in vitro derived blastocysts exhibited remarkable gene expression differences compared to competent in vivo ones, yet both resulted in pregnancy establishment. This expression difference between competent in vitro and in vivo derived embryos may indicate a possible cause for postnatal abnormalities sometimes observed in pregnancies established after transfer of in vitro derived embryos. Although, both competent in vivo and in vitro blastocysts are capable of inducing robust pregnancies, reports have shown that some abnormal outcomes after transfer of in vitro derived embryos, including postnatal consequences, prolonged gestations, fetal overgrowth such as the large offspring syndrome phenomena in cattle, longer hind legs in horses, metabolic disturbances including increased blood pressure, higher fasting glucose, and increased peripheral body fat deposits in childhood or adolescence in humans [51, 52]. Therefore, marked transcriptome differences between competent in vivo and in vitro derived blastocysts could partly be associated with postnatal abnormalities in the latter. However, this speculation requires further confirmation by analysing the gene expression outline of blastocyst biopsies ending up with abnormal postnatal consequences. One the other hand, differences in the gene expression patterns may not be stable. Thus, after transfer to recipients, the aberrant expression of these genes might only persist if the adverse environmental conditions persist, otherwise the expression patterns could return to the normal level after transfer to the physiological environment. But, there might be a considerable proportion of genes affected by the environmental condition which do not bear a consequence for an embryo’s ability to initiate a robust pregnancy and/or to term development.

Collectively, the results of the present study demonstrated, specific effects of the developmental environment on a bovine embryo’s gene expression outline by comparing embryos of comparable developmental capacity. Suboptimal developmental environments, as represented by the in vitro culture in the present study, caused aberrant expression of several gene clusters, especially those coding for ribosomal proteins, mitochondrial ribosomal proteins and NADH dehydrogenases. Noteworthy, especially transcripts related to ribosomal proteins were found to be preferentially expressed in inner cell mass cells compared to trophectoderm cells. Thus, the in vitro environment exhibits profound effects on biological processes associated with metabolism, ATP production and protein synthesis and molecular functions including oxidoreductase and cytochrome-c reductase activity. In the current study, while differentially expressed genes enriched in ribosomal proteins, mitochondrial ribosomal proteins and NADH dehydrogenase were not conflictive with pregnancy establishment at day 90.

### Molecular signatures predictive for embryos’ capability to initiate robust pregnancies

Transferable embryos are usually selected using morphological criteria in cattle as well as humans. Other non-invasive methods include evaluation of morphokinetic features, oxygen consumption and the presence of biochemical molecules within the embryo surrounding spent media [53]. However, non-invasive techniques are not consistent in producing the expected results, as these methods lack direct information about an embryo’s intrinsic characteristics. Consequently, embryo selection techniques based on its intrinsic quality using embryo biopsies approaches could provide detailed and reliable information about the molecular gene expression signature of the embryos. Therefore, assessing the intrinsic quality of an embryo would provide an alternative option to uncover the molecular mechanism governing successful embryonic development and to select embryos bearing high developmental potential. With this respect, identification of genes showing typical expression patterns reflective of high developmental competency could be an important step to identify molecular markers specifically associated with embryo developmental potential [54]. With this notion, in the current study, we also uncovered the transcriptome profiles of bovine embryos derived either from in vitro or in vivo comparing those with the ability to end up in a robust pregnancy against those lacking the ability to initiate early pregnancy using the EmbryoGENE microarray platform, which consists of about 45,000 probes. Accordingly, the present study identified about 700 and 218 annotated DEGs between competent in vivo derived embryos (CVO) and non-competent in vivo derived embryos (NVO) as well as competent in vitro derived embryos (CVT) and non-competent in vitro derived embryos (NVT), respectively. That suggests higher heterogeneities in the transcriptome profile due to developmental competence in embryos derived from in vivo compared to in vitro ones. When investigating the expression trend of differential expressed genes, most (82%) were upregulated in competent in vivo derived blastocysts compared to the non-competent ones. In contrast, about 89% of DEGs were down regulated in the competent in vitro derived embryos compared to their non-competent counterparts. That finding suggests that while increased transcriptional activity in the day 7 in vivo bovine embryos seems to go along with developmental competency, reduced transcriptional activity goes along with developmental competency of in vitro derived embryos. Conversely, higher homogeneity of in vitro derived embryos might be a consequence of higher selection pressure due to less favourable in vitro culture conditions. This assumption, however, is deduced based on overall expression patterns and thus further verification by independent studies is required.

In addition to exploring the global gene expression trends, identification of genes with high expression change and their relevant functions could help to identify transcripts associated with developmental competence. In line to this, 4.4 and 11.4% of all DEGs exhibited noticeable expression differences (≥ 3 fold changes) when comparing competent vs. non-competent in vivo derived embryos and competent vs. non-competent in vitro derived embryos, respectively (Supplemental Fig. 3). For instance, the expression of ribosomal proteins *(RPL34, RPS28, RPS24),* keratin 19 (*KRT19*), myosin light chain 7 *(MYL7),* glutaredoxin *(GLRX),* and *SERPINE1* mRNA binding protein 1 (*SERBP1)* was increased by 3.4–6.0 folds (*p* < 0.05) in competent vs. non-competent in vivo derived embryos (Fig. 2). Although characterizing the role of these genes in embryo development may require further studies, based on the previous findings it can be speculated that these genes could be involved in embryo development by regulating distinct cellular functions. For instance, previous reports have shown the potential role of *RPL34, RPS28* and *RPS24* in cell proliferation, cell cycle progression, and cell cycle processes [55–57] whereas *SERBP1* has been shown to play a role in the regulation of transcription, RNA metabolism and cell proliferation [58]. Therefore, higher expression of *RPL34, RPS28, RPS24, MYL7* and SERBP1 specifically due to higher developmental capacity in embryos derived in vivo*,* could implicate that the activity of these genes facilitate pregnancy establishment by controlling cell proliferation, cell cycle progression and regulation of transcriptional activities. On the other hand, nanog (*NANOG),* TNFAIP3 interacting protein 2 *(TNIP2),* branched chain aminotransferase 2 *(BCAT2),* FOS like 1, AP-1 transcription factor subunit *(FOSL1)* and actin beta (*ACTB)* were among the DEGs whose expression was reduced by 2.3–7.3 fold in competent compared to non-competent in vivo derived embryos. Among these, *NANOG* and *FOSL1* are believed to be involved in cell linage formation and defects during this critical developmental step could be a main cause of early pregnancy failure and disorders. For instance, higher activity of *NANOG*, a cell-specific gene and transcription factor related to pluripotency, is believed to be associated with an undifferentiated state of cells and could be involved in the maintenance of pluripotency in a dose-dependent manner [59]. *NANOG* is expressed in morula and inner cell mass (ICM) cells of human blastocysts whereas its expression is downregulated during mouse implantation [60, 61]. Similarly, *FOSL1*, a gene responsible for the development of trophoblast giant cells is involved in differentiation of embryonic stem (ES) cells to trophoblast lineage-like cells by activation of lineage-specific genes [62]. Lower expression of *NANOG* and Fosl1 in competent compared to non-competent in vivo derived embryos could therefore indicate a higher level of cell differentiation in the more competent in vivo derived embryos.

### Distinct gene cluster predictive for embryos’ capability to initiate robust pregnancies

In this study, we have identified several gene clusters encompassing groups of genes which potentially share a generalized function, being differentially expressed specifically due to developmental capacity of in vivo derived blastocysts while not observed to be differentially regulated in competent vs. non-competent in vitro derived blastocysts. Noteworthy, eukaryotic translation initiation factors such as *EIF1*, *EIF3, EIF3C, EIF3D, EIF3E, EIF3K, EIF4* and *EIF4E2* were among these clusters. Within these, the eukaryotic translation initiation factor 3 (eIF3) is the largest complex of the translation initiation factors comprised of thirteen subunits (eIF3a to eIF3m). Mice deficient in eIF3e were embryonically lethal. Depletion of eIF3e is suggested to cause reduced levels of eIF3a and eIF3c subunits, subsequently reducing cellular proliferation, suggesting an important role of eIF3e in embryonic development by affecting the global protein translation [63]. Likewise, the eukaryotic initiation factor 4E (eIF4E) which also showed increased expression in competent in vivo derived embryo within the current study is believed to be involved in mesoderm induction during embryogenesis [64], possibly by regulating cap-dependent translation [65]. Thus, we suggest that higher expression of these eukaryotic translation initiation factors might increase cellular proliferation and differentiation by regulating global protein translation turnover in competent in vivo derived blastocysts.

The ribosomal protein of small and large subunits (RPS3, − 6, − 8, − 21, − 24, − 28, RPL7, − 11, − 12, − 13. -15, − 23, − 24, −27A, − 30, − 31, − 34, − 35A, − 36, −37A, − 38, − 39) as well as mitochondrial ribosomal proteins (*MRPL10, MRPL16, MRPL42, MRPL52, MRPL55* and *MRPS18C*) were also among the gene clusters increased in competent in vivo derived embryos. Among these, *RPS3, − 6, − 8, − 21, − 24* and *RPS28* and large subunits such as *RPL11, − 13, − 15, − 23, − 24, −27A, − 30, − 31, − 35, − 36, −37A, − 38* were previously reported to be highly expressed in the human embryos at the blastocyst stage [66]. Bioinformatic analysis showed that these genes are structural constituents of the ribosomes and they are involved in ribosome pathways. Despite the fact that the exact role of ribosomal proteins in embryogenesis is not yet well described, it is well known, however, that small ribosomal proteins play a role in the initiation of translation while the large ribosomal proteins are involved in the formation of peptide bonds [67]. In line with this, a study conducted in HeLa cells indicated that *RPS6*, *RPS8*, *RPS24* and *RPS28* are required for initiation of the processing steps specific to the 18S pre-rRNA maturation pathway whereas *RPS3* is required for the nuclear and cytoplasmic maturation steps and for nuclear export of proteins [68]. Similarly, other studies also showed the role of *RPS6*, *RPS24*, *RPS28*, *RPL7*, *RPL11* and *RPL35A* in the production of mature ribosomes and functionally active polysomes [69], and specifically the role *RPL11* in embryonic development by regulating p53-dependent checkpoint responses [70]. Thus, increased expression levels of ribosomal proteins in competent in vivo derived blastocysts could reflect a more advanced status of differentiation or functionality in these embryos being beneficial for further development. In addition, further gene clusters associated with energy metabolism were also enriched in competent vs. non-competent in vivo derived blastocysts. These gene clusters include mitochondrial membrane ATP synthases (ATP5) subunits (*E, G1, G2, H, I, J, J2, L, O*), NADH: ubiquinone oxidoreductase core (NDUF) subunits (*S1, S2, S4, S5, S8, B8*), cytochrome c oxidase (COX) subunits (*5A, 6A1, 17A2, 6B1, 6B, 6C*), and aldehyde dehydrogenases (*ALDH16A1, ALDH3A2, ALDH7A1*). These gene clusters are involved in ATP production, molecular functions including oxidoreductase activity, electron transfer activity, NADH dehydrogenase activity and cytochrome-c oxidase activity specifically indicating the manifestation of higher ATP turnover due to high developmental competence in embryos derived in vivo. Furthermore, literature mining with respect to those individual genes showed that the ATP5E (the ε-Subunit of mitochondrial ATP synthase) gene could be involved in spindle orientation, nuclear divisions and centrosome positioning during embryonic divisions by increasing the ATP synthase activity [71]. Another subunit of *ATP5*, *ATP5H* and *ATP5B* have already been implicated in embryo implantation [72]. Likewise, the NADH: ubiquinone oxidoreductases such as *NDUFS1, NDUFS6* and *NDUFS8* are implicated in embryo development whereas cytochrome c oxidase subunits such as *COX5A, COX6B1* and *COX17* are implicated both in embryonic development and implantation [72]. Collectively, these results strongly indicate that the expression outline of gene clusters associated with mitochondrial functionality might be a good indicator for the capability of the in vivo derived embryo to end up in a robust pregnancy.

To get a more comprehensive overview about the function of these clusters of genes with respect to a blastocysts developmental competence, we analysed in which molecular pathways these clusters are involved. Strikingly, glycolysis, pyruvate metabolism and oxidative phosphorylation molecular pathways were enriched by these genes suggesting higher ATP accumulation in competent in vivo derived embryos using either aerobic or anaerobic energy providing pathways. Indeed, 86% of ATP production in bovine embryos during blastocyst formation is derived from oxidative phosphorylation [73]. This supports that ATP production of the blastocyst stage embryo is mainly derived from aerobic pathways and thus, higher expression of genes associated with ATP production in competent in vivo derived embryos could be a need for implantation and further development.

Collectively, the present study was able to unravel that compared to the non competent ones; competent in vivo derived embryos are characterized by higher expression of gene clusters including those involved in mitochondrial functions, ATP synthases, eukaryotic translation initiation factors, ribosomal proteins as well as NADH dehydrogenases. This can in turn indicate that competent in vivo derived embryos are characterized by upregulation of global protein translation turnover as well as ATP generating pathways. In contrast, compared to the none competent ones, in vitro derived competent embryos which usually have a low developmental capacity compared to in vivo due to non-physiological developmental environment, showed altered expression of genes involved in protein processing in the endoplasmatic reticulum, splicosome and ubiquitone mediated lysosome functions.

### Candidate gene expression signatures predictive for developmental capacity

Previously, Zoloni et al. [34] have most recently summarized the expression pattern of 19 DEGs related to developmental capacity of in vivo derived bovine embryos reported by Ghanem et al. [33], El-Sayed et al. [31] as well as others [32, 35]. Interestingly, the authors indicated that the expression levels of two candidate genes (*EEFA1A* and *KRT8*) were most likely associated with the pregnancy outcome of bovine blastocysts after transfer to recipients. In fact, previous reports [32, 33] were done only using microarray analysis consisting of few numbers genes and other studies [34, 35] were focused on female embryo competency in relation to the pregnancy outcome and embryos were categorized as competent when it induces pregnancy both at day 30 and day 60 while in the current study both in vitro and in vivo embryos were considered as developmentally competent if the pregnancy was maintained until day 90 and beyond. Despite of these differences, we have also merged the DEGs identified in CVO vs. NVO and CVT vs. NVT with that of all previous studies using [31–35] and were also able to identify these two transcripts among those genes being differentially expressed due to contrasting developmental capacity within the present study. Noteworthy, a total of 53 DEGs were commonly detected by our present study as well as by at least one previous one (Table 8). Among these, *EEFA1A, KRT8, ZNF281, GART, NMP1, TXN, PAG2G4* and *PLAC8* were differentially expressed between competent and non-competent blastocysts in at least three studies including the current one. Of these, *EEFA1A* and *KRT8* were identified in 5 out of 7 studies suggesting their involvement in pathways being highly correlated with developmental capacity. Moreover, 6 transcripts, namely *ZFN281, GART, EEF1A1, HSPA8, RALA* and *RER1* showed the same trend of expression for competent embryos in all studies analysing in vivo derived ones, whereas 8 transcripts, namely *NPM1, ENPEP, TXN, TPT1, RPL26, H2AFZ, DDX5* and *RPA3* showed the same trend of expression for competent in vitro derived embryos in all studies. Finally, 14 transcripts, namely *ZNF281, ZNFP36L1, YIPF5, TSMB4X, GART, FERMT2, CYP5A1, CTR9, CDYL, CCRN4L, PRKCQ, PFDN5, CTSZ* and *BNIP3* showed a similar expression trend both for competent in vivo and in vitro derived embryos. This suggested that these genes could be potentially the reflectors of the intrinsic quality of bovine blastocysts and their developmental competence to establish pregnancy. Conversely, 8 transcripts, namely *STAU2, SMAP, SLC36E3, PERP4, CSRP2, RPLP2, DSTN* and *CLIC1* showed a contradictory expression trend in the competent in vivo and competent in vitro derived embryos, implicating contrasting molecular needs for competent bovine embryos derived from contrasting developmental environments.

**Table 8:**
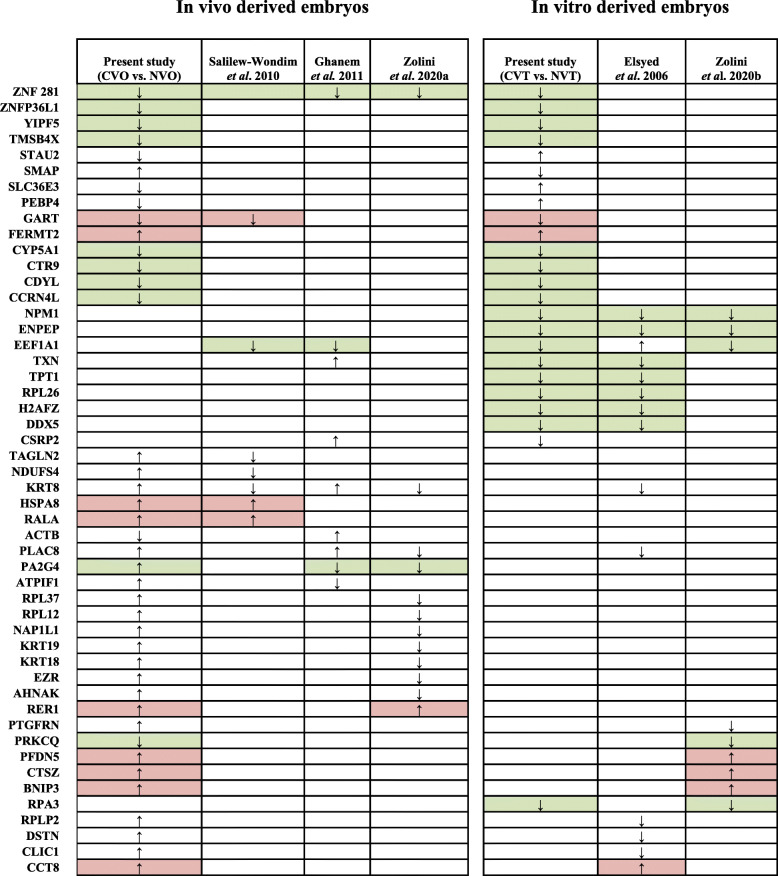
Molecular signatures of in vivo and in vitro derived embryos correlated with developmental capacity

Symbols, **↑** and ↓ indicate up and downregulated genes in competent blastocysts compared tovnone-competent ones.

### Do gene expression signatures reflective for environmental conditions or predictive for developmental capacity resemble gene expression of either ICM or TE cells?

Developmental competency of a given embryo in general depends on its ability to segregate into two main cell linages, the inner cell mass (ICM) and the trophectoderm (TE). This may lead to the hypothesis that preferential enrichment of genes reflective for developmental capacity in ICM or TE cells may determine the fate of the blastocyst towards establishment of a robust pregnancy. Indeed, previous reports have shown differential expression of genes between ICM and TE cells in mouse [74], human [75] and bovine embryos [42, 43, 76, 77]. Thus, analysis those distinct molecular signatures of the embryo which are predictors of developmental capacity in the cell lineages could be essential to generate basic knowledge to enlighten our understanding about the developmental capacity of bovine embryos. Also, the preferential expression of genes predicting developmental capacity in either ICM or TE cells could give an indication on whether one distinct cell lineage of the blastocyst (ICM or TE) is predominately fraught with problems during early development. Therefore, we superimposed those DEGs identified to be predictive for developmental competence in the current study with comparative transcriptome profiles of the ICM and the TE of in vivo and in vitro derived bovine blastocysts reported by Hosseini et al. [42] and Ozawa et al. [43], respectively. Of high interest, several genes that were differentially expressed between the competent and non-competent in vivo derived embryos were also differentially expressed between the ICM and TE. However, there was no indication for a preferential expression of genes predictive for developmental capacity in either ICM or TE cells considering in vivo and in vitro derived bovine embryos. Consequently, the present study did not identify one cell lineage prone to developmental problems. In contrast, gene expression signatures specifically correlated with developmental capacity seem to be distributed equally over both compartments.

## Conclusion

In this study, we outlined effects of different developmental environments as well as contrasting developmental capacities of bovine embryos on a gene expression outline. The results of the present study unravelled that competent in vivo derived embryos, serving as gold standards, are characterized by higher expression of genes in gene clusters involved in mitochondrial functions, ATP synthases, eukaryotic translation initiation factors and ribosomal proteins, indicating that competent in vivo derived embryos are characterized by upregulation of global protein translation turnover as well as ATP generating pathways. In contrast, in vitro derived embryos of low developmental capacity, showed adverse expression of genes involved in protein processing in the endoplasmatic reticulum, splicosome and ubiquitone mediated lysosome. Since the latter had developed within a non-physiological environment, the present study also demonstrated a specific effect of the developmental environment on a bovine embryo’s gene expression outline. To our knowledge, this is the first study in analysing environmental effects on bovine embryos by comparing embryos of comparable developmental capacity instead of using a pool of embryos consisting of different developmental capacity. Representing a main result, non-physiological developmental environments, as represented by the in vitro culture in the present study, cause aberrant expression of gene clusters especially coding for ribosomal proteins, mitochondrial ribosomal proteins and NADH dehydrogenases. Besides, the present study demonstrates that the in vitro environment exhibits down regulative effects on gene signatures, and also shows those gene signatures to be predictive for high developmental capacity of embryos developed in physiological environments. While differential expression of genes enriched in ribosomal proteins, mitochondrial ribosomal proteins and NADH dehydrogenase, as a reflection of non-physiological environments were not conflictive with pregnancy establishment at day 90..

Altogether, the present study provides a detailed inventory of differentially expressed candidate genes, gene signatures and pathways reflecting contrasting developmental environments as well as distinct gene signatures and pathways being predictive for future developmental capacity in bovine embryos. Noteworthy, the gene signatures reported to be predictive for developmental capacity by the present study are encompassing gene variants, alternative 3′UTR events and indel type splice variants for the first time. Of high importance, non-physiological culture environments were found to be reflected in differential expression of gene signatures, which were in turn predictive for low developmental capacities.

## Supplementary Information

**Additional file 1: Figure S1.** Molecular signatures reflecting environmental conditions in competent embryos. Volcano plot demonstrating differentially expressed genes between CVT and CVO blastocysts. Red and green dots indicate up and downregulated genes, respectively in CVO compared to NVO blastocysts.

**Additional file 2: Figure S2.** Molecular pathways significantly enriched by differentially expressed genes specifically modulated by environmental conditions in competent embryos (CVT vs. CVO). Terms on the left represent pathway modules (A) and terms on the right hand represent specific particular pathways enriched by differentially expressed genes (B). NAFLD: Non-alcoholic fatty liver disease.

**Additional file 3: Figure S3.** Relative proportion of differential expressed genes (*p* < 0.05) within distinct fold change categories in competent vs. non-competent in vivo derived embryos (CVO vs. NVO) and competent vs. non-competent in vitro derived embryos (CVT vs. NVT), respectively.

**Additional file 4: Table S1.** The list of differentially expressed genes between CVO and NVO. **Table S2.** The list of differentially expressed genes between CVT and NVT. **Table S3.** Significant molecular functions enriched by differentially expressed genes between CVT and NVT. **Table S4.** DEGs common to current study (CVO vs NVO) and Hosseini et al. 2015 (ICM vs .TE) and Ozawa et al. 2012 (TE vs .ICM). **Table S5.** DEGs common to current study (CVT vs NVT) and Hosseini et al. 2015 (ICM vs .TE) and Ozawa et al. 2012 (TE vs .ICM). **Table S6.** List of differentially expressed genes between CVT vs. CVO. **Table S7.** List of differentially expressed probes in CVT vs. CVO representing the 3′-UTR variants. **Table S8.** Molecular fuctions overrepresented by DEGs idnetfied in CVT vs. CVO. **Table S9.** Molecular functions overrepresneted by genes differentially expressed between CVT and CVO. **Table S10.** DEGs common to current study (CVT vs CVO) and Hosseini et al. 2015 (ICM vs .TE) and Ozawa et al. 2012 (TE vs .ICM).

**Additional file 5: Table S11.** Gene variants differential expressed between CVT and CVO groups.

## Data Availability

All data used and/or analyzed during the present study are available from the corresponding author if requested.

## Abbreviations

CVO: Competent in vivo blastocysts that resulted in pregnancy
CVT: Competent in in vitro blastocysts that resulted in pregnancy
NVO: Non competent in vivo blastocysts that resulted in no pregnancy
NVT: Non competent in vitro blastocysts that resulted in no pregnancy
DEGs: Differentially expressed genes
