# Supplementary figures and images for: The global gene expression outline of the bovine blastocyst: reflector of environmental conditions and predictor of developmental capacity
Source: BMC Genomics. 2021 Jun 3;22:408. doi: 10.1186/s12864-021-07693-0 (PMC8176733; doi:10.1186/s12864-021-07693-0)

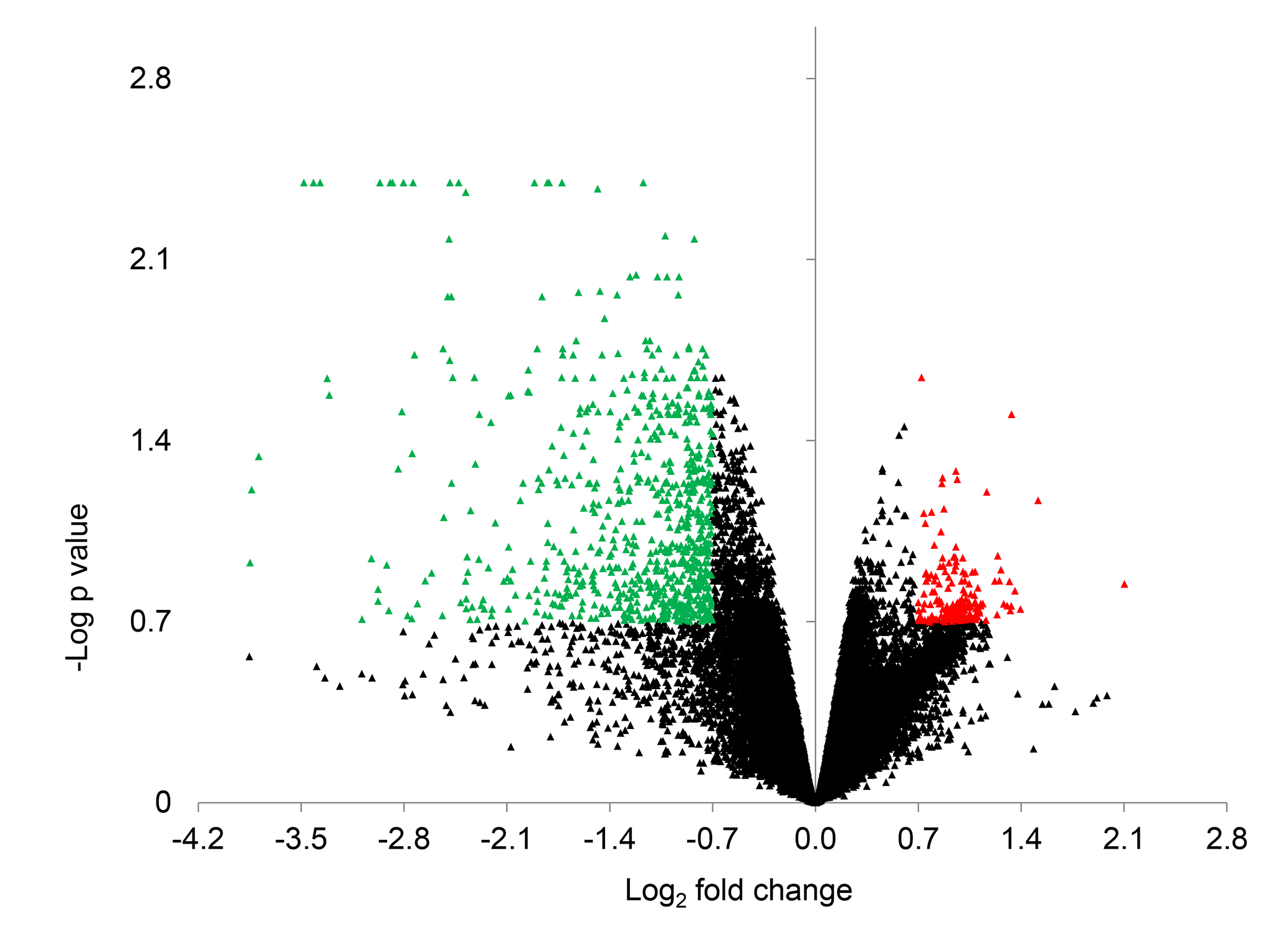

Supplement: Supplementary file 1 — Additional file 1: Figure S1. Molecular signatures reflecting environmental conditions in competent embryos. Volcano plot demonstrating differentially expressed genes between CVT and CVO blastocysts. Red and green dots indicate up and downregulated genes, respectively in CVO compared to NVO blastocysts. [file 12864_2021_7693_MOESM1_ESM.tif]

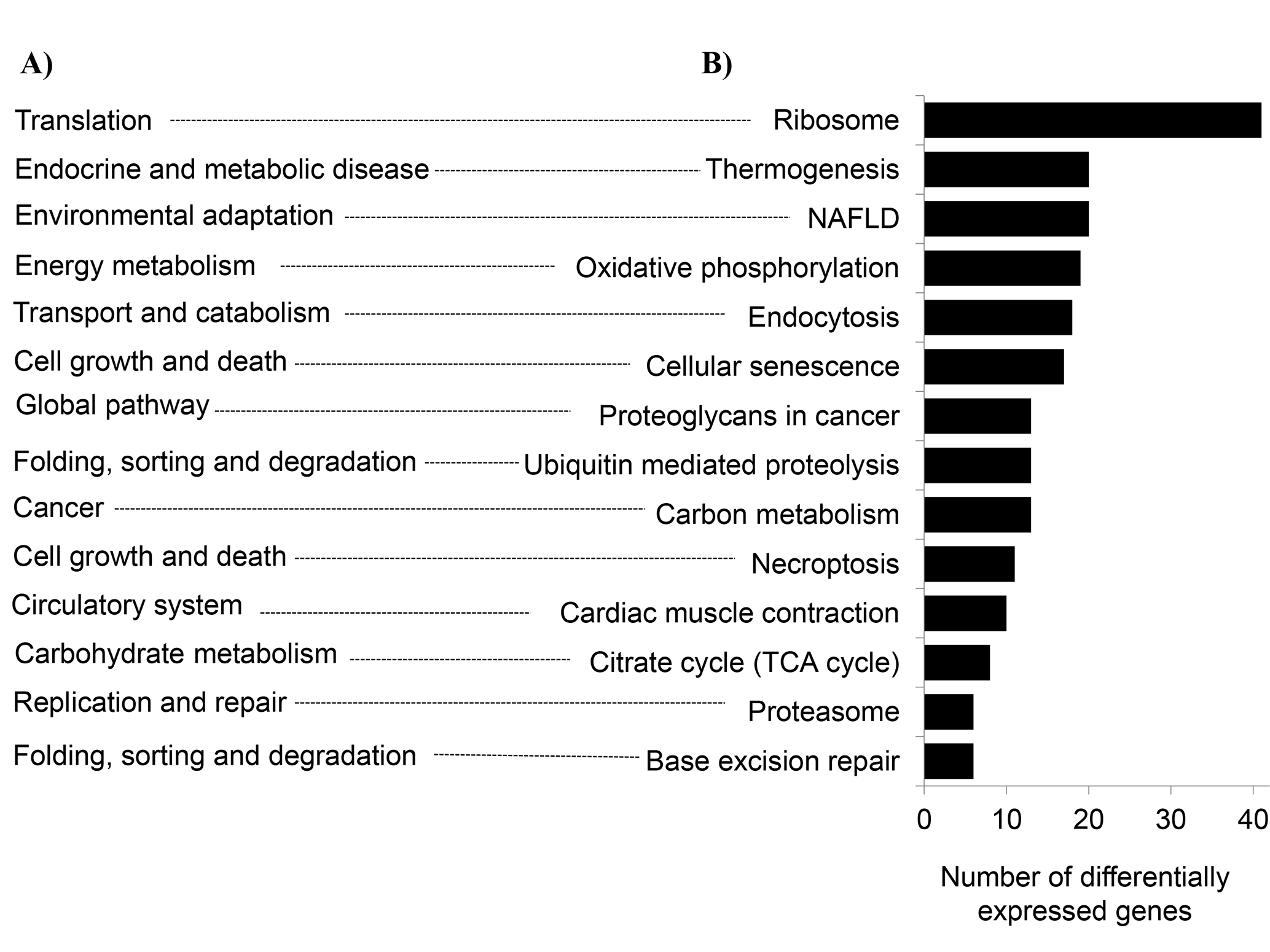

Supplement: Supplementary file 2 — Additional file 2: Figure S2. Molecular pathways significantly enriched by differentially expressed genes specifically modulated by environmental conditions in competent embryos (CVT vs. CVO). Terms on the left represent pathway modules (A) and terms on the right hand represent specific particular pathways enriched by differentially expressed genes (B). NAFLD: Non-alcoholic fatty liver disease. [file 12864_2021_7693_MOESM2_ESM.tif]

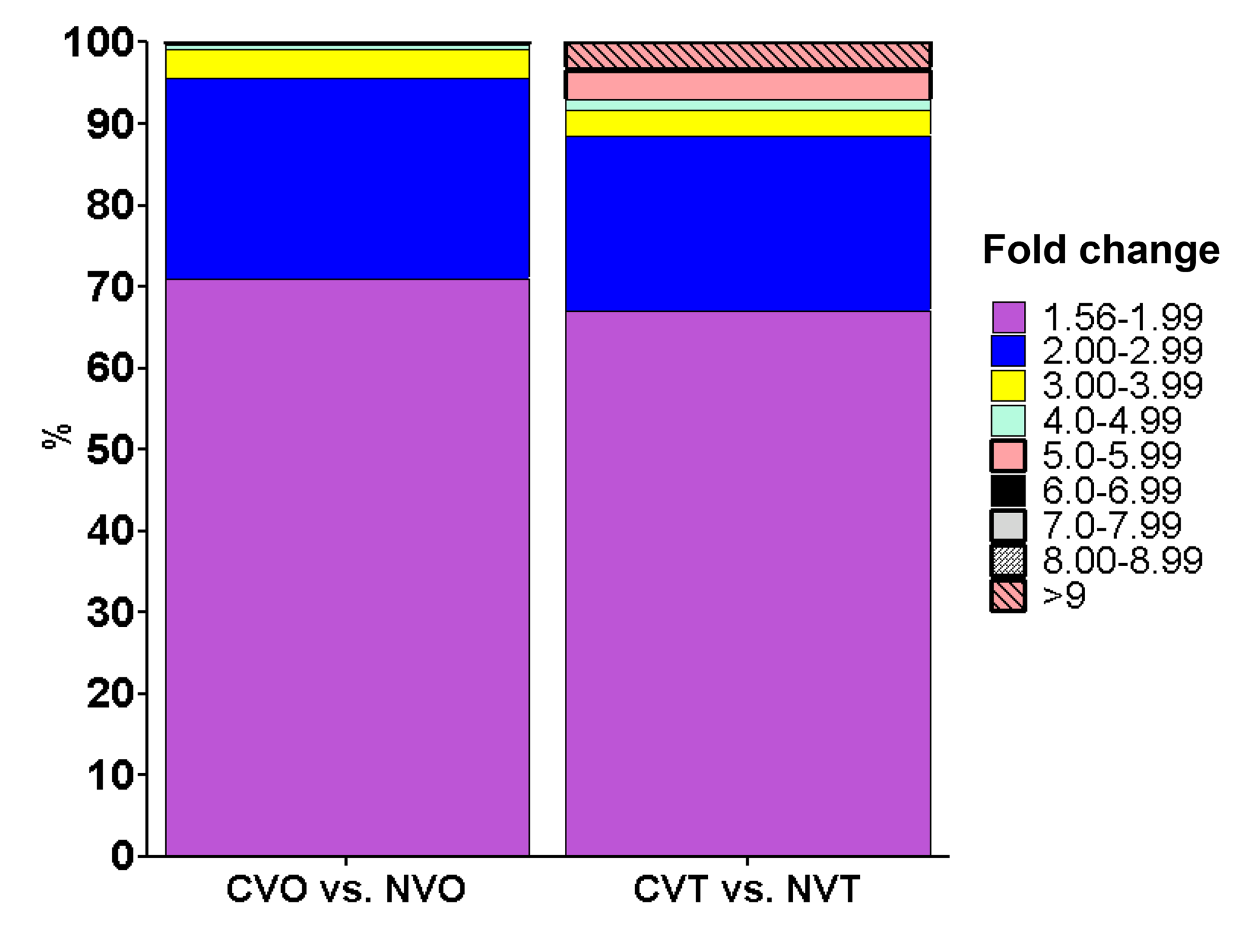

Supplement: Supplementary file 3 — Additional file 3: Figure S3. Relative proportion of differential expressed genes (p < 0.05) within distinct fold change categories in competent vs. non-competent in vivo derived embryos (CVO vs. NVO) and competent vs. non-competent in vitro derived embryos (CVT vs. NVT), respectively. [file 12864_2021_7693_MOESM3_ESM.tif]
