# Supplementary material for: The global gene expression outline of the bovine blastocyst: reflector of environmental conditions and predictor of developmental capacity
Source: BMC Genomics. 2021 Jun 3;22:408. doi: 10.1186/s12864-021-07693-0 (PMC8176733; doi:10.1186/s12864-021-07693-0)
Supplement: Supplementary file 5 — Additional file 5: Table S11. Gene variants differential expressed between CVT and CVO groups. [file 12864_2021_7693_MOESM5_ESM.docx]

**Table S11:** Gene variants differential expressed between CVT and CVO groups

| **Probe ID** | **Target location** | **Gene symbol** | **Description** | **Expression** |
| --- | --- | --- | --- | --- |
| EMBV3_36169 | NM_001105501 | UBFD1 | Ubiquitin family domain containing 1 | **↓** |
| EMBV3_27999 | NM_001105501:380^970 | UBFD1 | Ubiquitin family domain containing 1 | **↑** |
| EMBV3_15992 | NM_001105622:274^624 | TWISTNB | TWIST neighbor | **↓** |
| EMBV3_39846 | NM_205772:98^184 | SPG21 | Spastic paraplegia 21 (autosomal recessive, Mast syndrome) | **↑** |
| EMBV3_22048 | NM_174659 | SLC25A5 | Solute carrier family 25 (mitochondrial carrier; adenine nucleotide translocator), member 5 | **↓** |
| EMBV3_26357 | NM_174659:703^742 | SLC25A5 | Solute carrier family 25 (mitochondrial carrier; adenine nucleotide translocator), member 5 | **↓** |
| EMBV3_29618 | XM_002690797:448^620 | RTF1 | Rtf1, Paf1/RNA polymerase II complex component, homolog (S. cerevisiae) | **↓** |
| EMBV3_06189 | NM_174379:713^759 | RPSA | Ribosomal protein SA | **↓** |
| EMBV3_07745 | NM_174379 | RPSA | Ribosomal protein SA | **↓** |
| EMBV3_32650 | NM_001012682:838^949 | RPLP0 | Ribosomal protein, large, P0 | **↓** |
| EMBV3_01473 | NM_001012682:853^948 | RPLP0 | Ribosomal protein, large, P0 | **↓** |
| EMBV3_16252 | NM_001012682:853^949 | RPLP0 | Ribosomal protein, large, P0 | **↓** |
| EMBV3_07077 | NM_001012682:603^943 | RPLP0 | Ribosomal protein, large, P0 | **↓** |
| EMBV3_20357 | NM_001012682:611^945 | RPLP0 | Ribosomal protein, large, P0 | **↓** |
| EMBV3_16518 | NM_001012682:600^954 | RPLP0 | Ribosomal protein, large, P0 | **↓** |
| EMBV3_14209 | NM_001012682 | RPLP0 | Ribosomal protein, large, P0 | **↓** |
| EMBV3_04071 | NM_001075581:182^330 | RPL11 | Ribosomal protein L11 | **↓** |
| EMBV3_22780 | XM_002694590:137^399 | PTPN11 | Protein tyrosine phosphatase, non-receptor type 11 | **↑** |
| EMBV3_16342 | XM_002694966:496^634 | PPP1R14A | Protein phosphatase 1, regulatory (inhibitor) subunit 14A | **↓** |
| EMBV3_14540 | NM_001101225:426^566 | POLE4 | Polymerase (DNA-directed), epsilon 4 (p12 subunit) | **↓** |
| EMBV3_12712 | NM_001034384:651^728 | NOL7 | Nucleolar protein 7, 27kDa | **↓** |
| EMBV3_04020 | NM_174819:167^689 | NDUFS3 | NADH dehydrogenase (ubiquinone) Fe-S protein 3, 30kDa (NADH-coenzyme Q reductase) | **↓** |
| EMBV3_28321 | NM_175787:271^363 | NDUFB6 | NADH dehydrogenase (ubiquinone) 1 beta subcomplex, 6, 17kDa | **↓** |
| EMBV3_41338 | XM_002699027:382^483 | NAALAD2 | N-acetylated alpha-linked acidic dipeptidase 2 | **↑** |
| EMBV3_35847 | NM_001076018:227^306 | MTHFD1L | Methylenetetrahydrofolate dehydrogenase (NADP+ dependent) 1-like | **↓** |
| EMBV3_13857 | NM_001046508 | MRPS18C | Mitochondrial ribosomal protein S18C | **↓** |
| EMBV3_27125 | NM_001046508:169^252 | MRPS18C | Mitochondrial ribosomal protein S18C | **↓** |
| EMBV3_24774 | NM_001035078:448^513 | MRPS11 | Mitochondrial ribosomal protein S11 | **↑** |
| EMBV3_18927 | NM_001080730:928^975 | MRPL39 | Mitochondrial ribosomal protein L39 | **↓** |
| EMBV3_05811 | XM_002692789 | MGC148714 | Similar to Cytochrome c oxidase polypeptide VIc-2 | **↓** |
| EMBV3_08656 | XM_002692789:248^375 | MGC148714 | similar to Cytochrome c oxidase polypeptide VIc-2 | **↓** |
| EMBV3_35030 | NM_001144098:172^250 | MEP1B | Meprin A, beta | **↑** |
| EMBV3_33464 | XM_002697647:677^931 | MBP | Myelin basic protein | **↑** |
| EMBV3_31117 | NM_001143862:236^325 | LSM7 | LSM7 homolog, U6 small nuclear RNA associated (S. cerevisiae) | **↓** |
| EMBV3_07099 | XM_002699749:287^754 | LOC618023 | Similar to mCG1035526 | **↓** |
| EMBV3_04189 | XM_002694113:286^391 | LOC616065 | Similar to 60S ribosomal protein L22 (Heparin binding protein HBp15) | **↓** |
| EMBV3_31480 | XM_002689372:572^809 | LOC510390 | Similar to Vacuolar ATP synthase subunit S1 precursor (V-ATPase subunit S1) (V-ATPase S1 accessory protein) (V-ATPase Ac45 subunit) (C7-1 protein) | **↑** |
| EMBV3_14709 | XM_002685968:584^721 | LOC100336730 | Myocyte enhancer factor 2D-like | **↑** |
| EMBV3_32750 | NM_001014953:658^797 | LMAN2L | Lectin, mannose-binding 2-like | **↑** |
| EMBV3_18426 | NM_001035370:57^566 | KLHDC8B | Kelch domain containing 8B | **↑** |
| EMBV3_34242 | NM_001034336:266^612 | ERH | Enhancer of rudimentary homolog (Drosophila) | **↓** |
| EMBV3_26896 | XM_002688873:867^940 | ELAVL3 | ELAV (embryonic lethal, abnormal vision, Drosophila)-like 3 (Hu antigen C) | **↑** |
| EMBV3_03176 | NM_001075752:68^417 | EIF4H | Eukaryotic translation initiation factor 4H | **↑** |
| EMBV3_36018 | NM_001033763:224^944 | DNAJB1 | DnaJ (Hsp40) homolog, subfamily B, member 1 | **↑** |
| EMBV3_38045 | NM_001038183 | CSRP2 | cysteine and glycine-rich protein 2 | **↓** |
| EMBV3_37652 | NM_001038183:283^506 | CSRP2 | Cysteine and glycine-rich protein 2 | **↓** |
| EMBV3_42215 | NM_175807:98^187 | COX7A2 | Cytochrome c oxidase subunit VIIa polypeptide 2 (liver) | **↓** |
| EMBV3_03749 | NM_001077831:264^339 | COX6A1 | Cytochrome c oxidase subunit VIa polypeptide 1 | **↓** |
| EMBV3_34344 | NM_001002891:521^562 | COX5A | COX5A protein | **↓** |
| EMBV3_21683 | NM_001002891 | COX5A | COX5A protein | **↓** |
| EMBV3_08339 | NM_001002891:400^444 | COX5A | COX5A protein | **↓** |
| EMBV3_23073 | NM_001075221:198^274 | C19H17orf37 | MGC142432 chromosome 17 open reading frame 37 ortholog | **↑** |
| EMBV3_32783 | NM_001105436:700^814 | AUH | AU RNA binding protein/enoyl-Coenzyme A hydratase | **↑** |
| EMBV3_06291 | NM_001113719:133^249 | ATP5J2 | ATP synthase, H+ transporting, mitochondrial F0 complex, subunit F2 | **↓** |
| EMBV3_28386 | NM_001113719:163^210 | ATP5J2 | ATP synthase, H+ transporting, mitochondrial F0 complex, subunit F2 | **↓** |
| EMBV3_12042 | NM_001075136:123^864 | ATP5C1 | ATP synthase, H+ transporting, mitochondrial F1 complex, gamma polypeptide 1 | **↓** |
| EMBV3_07020 | XM_002689954:572^724 | ASTN2 | Astrotactin 2 | **↑** |
| EMBV3_01211 | NM_001075813:596^942 | AIMP2 | MGC152188 aminoacyl tRNA synthetase complex-interacting multifunctional protein 2 | **↑** |

Arrows ↑ and ↓ indicate up and down regulation in CVT compared to CVO blastocysts
